# Heat stress reproportions distinct metabolic sub-populations of coral-algal endosymbionts

**DOI:** 10.1093/ismeco/ycag099

**Published:** 2026-04-20

**Authors:** Daniel A Nielsen, Philip Heraud, Trent D Haydon, Katherina Petrou

**Affiliations:** School of Life Sciences, University of Technology Sydney, Ultimo, NSW, Australia; School of Chemistry, Monash University, Clayton, VIC, Australia; Center for Genomics and Systems Biology, New York University Abu Dhabi, Abu Dhabi, UAE; School of Life Sciences, University of Technology Sydney, Ultimo, NSW, Australia

**Keywords:** coral, heat stress, endosymbiosis, metabolome, microspectroscopy

## Abstract

Increasing occurrences of mass heat-induced coral bleaching around the globe have propelled research effort into enhancing coral resilience. Yet, significant progress in this space is hampered by an incomplete understanding of the inter-cellular processes sustaining the delicate, animal-algal symbiosis that underlie coral health. To elucidate links between changes in the symbiotic algal physiology and bleaching, we measured metabolic fingerprints of >1500 endosymbiotic algal cells from four coral species exposed to control and heat-stress conditions. We detected four co-occurring endosymbiont metabolomes based on spectral features, finding strong parallels across species. Clear temporal shifts in the dominance of each metabolome helped link metabolic profiles to cellular physiological states within the coral colony endosymbiotic landscape. We found two profiles common to healthy endosymbionts and two profiles reflective of physiological stress. In the absence of heat-stress, the most prevalent metabolic profiles were differentiated by high protein, high nucleic acid content and low carbon (lipid and/or carbohydrate) content. Whereas during late-stage bleaching, the dominant metabolic profiles exhibited comparatively low protein, but high carbon content. This work has uncovered the existence of endosymbiont metabolic sub-populations within coral colonies and shown their dynamic yet predictable reproportioning during heat stress conditions across different coral species. In identifying a physiological cascade of single-cell metabolomes in response to heat stress, this research highlights promising metabolic markers for detecting the onset of heat stress and dysbiosis within individual endosymbiotic coral cells.

## Introduction

Coral reefs form one of the most diverse ecosystems on the planet [1]. As foundation species, reef-building (scleractinian) corals are essential to the health and functioning of the reef ecosystem, providing a valuable food resource and habitat through their structural complexity. Scleractinian corals are comprised of an animal host (phylum *Cnidaria*) and an intracellular microalga (family *Symbiodineaceae*) [2] that live in a mutually beneficial symbiotic relationship based on nutrient exchange; the host providing inorganic nutrients [3] and in return, the symbiont delivering energy rich photosynthetic products such as glycerol and lipids to the host [4]. The coral host may also obtain nutrients through directly feeding on the symbionts, effectively farming the algae as a long-term nutrient supply [5, 6]. This relationship between host and alga has enabled corals to proliferate and thrive for millions of years in the otherwise nutrient poor waters of the tropics. Today, global warming is threatening coral reefs across the globe [7], putting much of the ocean’s biodiversity at risk [1, 8].

Warming sea temperatures can result in the loss of algal symbionts from the coral tissue and ultimately result in the death of the coral due to starvation. The cellular mechanism of this physiological response, known as “bleaching” because of the resulting tissue colour change, has been the subject of intense research for decades. Owing to the complexity of the coral symbiotic system and the enormous genotypic diversity across both corals and symbionts, progress in our understanding of the bleaching mechanism has been slow and many of the functional levers in the symbiosis are unresolved [9]. As a result of increasing occurrence of extreme ocean warming events, coral reefs are now bleaching with a frequency and severity that is almost certain to impact their long-term persistence on our planet, with even bleaching-resistant corals being impacted by recurrent heatwaves [10]. For this reason, efforts are mounting to find ways to protect corals from the detrimental effects of a warming planet. These include, surveying for resilient corals, genetic modification, ecosystem management and thermal priming (e.g. [11–14]). Fundamental to any such endeavours, however, is the need to understand the biology of the coral itself.

With the advancement of deep sampling “omics” techniques, our understanding of the coral and symbiont physiology is improving [9], but a key limitation to these and other techniques is the need for bulk tissue sampling strategies. Recently, the first cell-atlas of a stony coral, *Stylophora pistillata*, was published [15], revealing >40 cell types across the life cycle of the coral animal. Of these 40 cell types, only one forms a symbiotic bond with algae and thus is directly relevant to the study of the symbiosis. To complicate the matter, corals sometimes harbour multiple species of algae at varying proportions, many of which have been shown under cultured conditions to differ in their response to environmental stressors [16]. Furthermore, as each symbiotic algal cell is an individual organism, residing in a discrete location of the coral tissue at discrete timepoints in their life cycles, multiple physiologies must exist within the coral tissue at any given time. Indeed, a previous study of metabolic profiles of individual algal symbiont cells found significant variability within colonies, both in terms of biomolecular composition and photophysiological states [17]. This diversity of symbiont metabolic states may partly be driven by differing life-stages, but also by differing conditions across the coral tissue [18]. As such, while excellent progress has been and continues to be achieved through bulk metabolomics (e.g. [19]), proteomics (e.g. [20, 21]), and transcriptomic analyses (e.g. [22]), single-cell techniques are integral to deepen our understanding of the cellular mechanisms behind the coral symbiosis and the pathophysiological response of the bleaching process.

In recent years, evidence has been mounting that the initiation of the breakdown of the symbiosis between the coral host and the algal symbiont is linked to a destabilization of the nutrient exchange (i.e. nitrogen and carbon) between the two partners: According to this novel hypothesis, metabolic stress as a result of heat increases protein catabolism in the host, which in turn increases nitrogen availability to the symbiotic alga, causing the alga to retain more photosynthate for growth and thus ultimately starving the host [22, 23]. In a follow up study, Rädecker et al. confirmed that the carbon and nitrogen flow between symbiont and host were interdependent, with the host assimilating more nitrogen when receiving carbon from the symbiont, thus limiting nitrogen supply to the symbiont and forcing continued carbon exudation [24]. Cellular changes due to the shifts in carbon and nitrogen content proposed by this sequence of events would likely manifest as distinct features in the metabolome of the algal cells. However, given the inherent diversity (species, cell type, and physiology) of cells that make up the coral, investigations would need to account for cell-specific metabolic shifts. Using infrared microspectroscopy and machine learning, we resolved the biomolecular profiles of individual algal endosymbiont cells across different coral species and conditions. Using the spectral fingerprints of biologically important compounds (such as lipids, proteins, carbohydrates, and nucleic acids) we provide a comprehensive metabolic overview of each cell and explore the relationship between heat stress and the proportion of distinct metabolic profiles in the algal populations across time.

## Materials and methods

### Coral collection and experimental design

Eight individual colonies for each of four scleractinian corals, *Pocillopora damicornis* (PD)*, S. pistillata* (SP)*, Acropora millepora* (AM), and *Acropora aspera* (AA), were collected from the reef flat of Heron Island, Great Barrier Reef, Australia in November 2018. Coral tips (~4 cm each) were excised and kept in shaded (50%) flow through tanks for five days, held upright by pegs, before commencement of thermal stress. The tips were subsequently allocated to one of two treatments, one set (from *n* = 4 colonies, unpaired design) maintained at ambient summer water temperature (control; μ = 28°C) throughout the experiment, while tips from the other colonies (*n* = 4) were exposed to temperature increases of 1°C per day until reaching 30°C and then 0.5°C per day until reaching a maximum of 31°C. Temperature was allowed to oscillate over day/night cycles, and the incubations were maintained for 2 weeks. At the time of sampling, the waters in the Heron Island lagoon had increased in temperature above normal (up to ~29°C), and some bleaching of corals had occurred prior to the experiment (visual colour reduction). This was reflected in a general recovery of symbiont cell density per area across corals in the first week of incubation before exposure to severe heat stress (Supplementary Fig. S2).

### *Physiological and morphological condition* in vivo

Physiological condition of symbiont cells (*in-hospite*) was assessed in vivo by chlorophyll *a* fluorescence using a pulse amplitude modulated fluorometer (Diving PAM, Walz GmbH, Effeltrich, Germany). Measurements were made twice daily, at midday and half an hour after sunset to determine light-adapted effective quantum yield of PSII (ΔF/F_M_’) and the dark-adapted maximum quantum yield of PSII (F_V_/F_M_), respectively [25].

### Symbiont cell density

Coral fragments from each colony of each species were collected daily for determination of symbiont cell density. Briefly, tissue was released from coral fragments using a sodium hydroxide digest method reported previously [26]. The resulting slurry was centrifuged, washed in filtered seawater and homogenized and symbiont cell concentration determined by manual counting using a haemocytometer (*n* = 6 counts per sample). Cell densities were normalized to surface area of the respective coral fragment, which was determined using a standardized wax technique (Stimson & Kinzie, 1991).

### Endosymbiont extraction

Intact endosymbiotic algal cells were extracted from each coral colony as described by Nielsen et al. 2018 [27]. Extracted cells were re-suspended in 100 μL FSW and preserved in 2% formalin. See supplementary methods for further details.

### Symbiont classification via ITS2 sequencing and analysis

DNA was extracted from isolated symbionts at T0 (see Symbiont cell density) using DNeasy Blood and Tissue kit (QIAGEN, Hilden, Germany) according to manufacturer’s specifications. To assess the Symbiodiniaceae community composition, extracted DNA was amplified using ITSintfor2 and ITS2-reverse primers [28, 29] with attached Illumina adaptors in Hot Start High-Fidelity 2x Master Mix (New England Biolabs, Ipswich, MA, USA). Indexed amplicons were pooled and sequenced on the Illumina MiSeq platform (2x 300 bp) at the Australian Genomic Research Facility (Victoria, Australia). Resulting data were processed using the SymPortal analytical framework, and paired-ended sequences were quality controlled using mothur 1.43.0 [30], the blast+ suite of executables [31], and minimum entropy decomposition [32]. See supplementary methods for further details.

### Synchrotron-based Fourier transform infra-red microspectroscopy

Synchrotron Fourier Transform Infra-Red (FTIR) microspectroscopy was performed on three sets of samples for each species at the Australian Synchrotron IRM beamline. Samples from three time points were selected for each species independently to represent pre-stress (Days 9 and 10; no apparent cell loss or reduction in F_V_/F_M_), early-stress (Days 14 and 15; just before commencement of cell loss and a decline in F_V_/F_M_ in treatment colonies) and late bleaching states (Day 19; maximum cell loss, significant decline in F_V_/F_M_ [18%-53%]). Measurements were made on hydrated cells through a CaF_2_ window. Each endosymbiotic algal cell was visually identified through the microscope at 400 times magnification (Supplementary Fig. S3). Only algal cells visibly encased in a host cell membrane were measured, ensuring only endosymbiotic algal cells were included in the study of. The presence of the host cell was considered negligible compared to the absorbance of the algal cell (see [17] for further information). Spectra were acquired over the measurement range 4000-800 cm^−1^ with a Vertex 80v FTIR spectrometer (Bruker Optics, Ettlingen, Germany) in conjunction with an IR microscope (Hyperion 3000, Bruker) fitted with a liquid nitrogen cooled mercury cadmium telluride detector. Co-added interferogram scans (*n* = 32-64) were collected at a 4 cm^−1^ wavenumber resolution. Spectral measurements were made in transmission mode using an aperture size of 4 μm x 4 μm. Spectral acquisition and instrument control were performed using Opus 6.5 software (Bruker). See supplementary methods for further details.

### Spectra pre-processing

Spectra were exported for analysis using R (v.4.3.1. [33]). Each spectrum was smoothed (4 pts either side) and second derivative transformed using the Savitzky–Golay filter from the R package prospectr [34], accounting for baseline slope and offset, and revealing bands otherwise obscured in the raw unprocessed spectra. The spectra were then reduced to spectral regions containing the major biological bands (3050–2800 and 1800–1000 cm^−1^), mean-centred and normalized using the standard normal variate method to account for differences in pathlength. Due to almost total absorbance by liquid water in the region containing the amide I peak (1673–1615 cm^−1^), this wavenumber range was removed from all analyses. Principal component analyses (PCA) outlier detection and visual inspection were used to identify spectra with poor signal to noise ratio and those affected by water vapour contamination or scattering artefacts (total of 20 spectra), which were subsequently removed from the dataset.

### Classification of cell types: Cluster analysis

To detect spectra representing cells in different metabolic states, spectral clustering was employed using the Spectrum function in the R package Spectrum [35]. Spectral clustering is a non-parametric clustering method that uses heuristics based on graphs and connectivity and can identify clusters of arbitrary shapes and is robust to noise and outliers [36]. Data from all days and treatments were analysed together for each species to ensure that the underlying assumptions for cluster generation were identical across all samples. Before analysis, zero and low variance wavenumbers were identified using the function nearZeroVar from the R package “caret” [37] and excluded. To further reduce noise and the influence of co-variates, the data were condensed into principal components using the base R function “prcomp” with scaling set to “TRUE.” The number of PCs to include for analysis was initially evaluated using the function findPC [38] with mean aggregation, and a heuristic approach was employed to select the number of clusters for each species. See supplementary methods for further details.

### Peak finding and classification

A total of 33 peaks were identified from the averaged spectral data for each species and cluster (Table S2). The wavenumber range for each peak was checked for each species and the widest range included for automatic peak finding within each spectrum, allowing for differing location of maxima and minima due to peak shifting. Each peak area was calculated by summing the normalized absorbance value across the peak range. Peaks were classified based on known wavenumber ranges from the literature (Table S2) and evaluated from PC1, 2, and 3 loadings from PCAs based on the processed spectral data. Of the 33 included peaks, 7 could not be classified unambiguously into a biomolecular group and were therefore termed “Not classified.”

**Figure 1:**
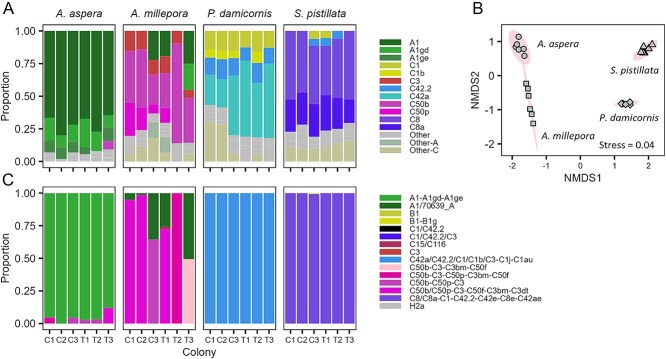
Symbiont community composition across coral species. (A) % contribution of the dominant symbiont clades (ITS2) at T0, with “C” designating control colonies and “T” designating treatment colonies and numbers indicating the replicate colony, (B) non-metric multidimensional scaling (nMDS) of symbiont community composition (ANOSIM R > 0.99, *P* < 0.0001). Shading indicates 95% confidence intervals. (C) ITS2 profiles based on grouping of closely related genome variants.

**Figure 2:**
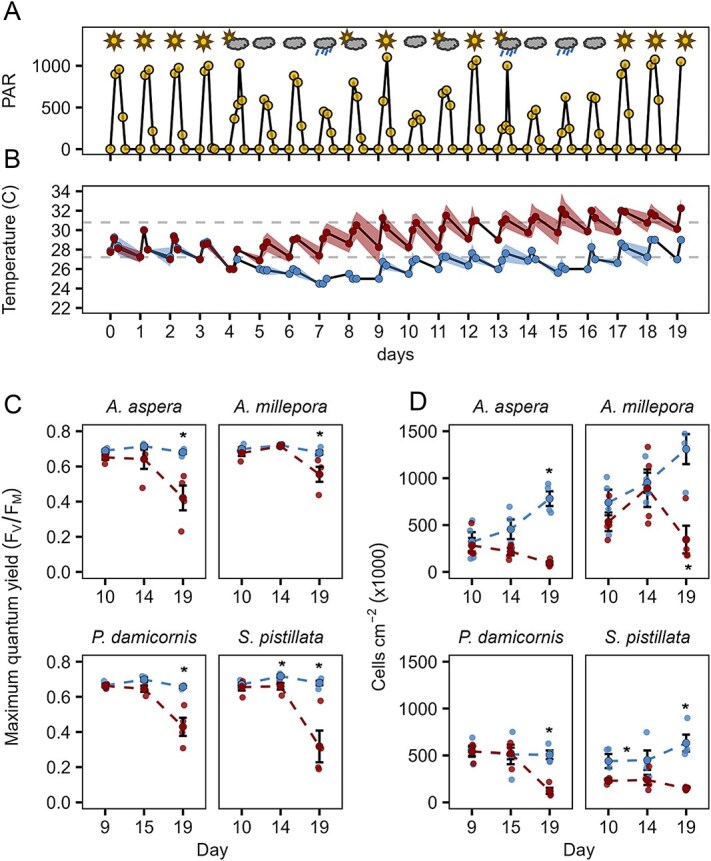
Environmental conditions and coral physiological parameters. (A) Daily photosynthetically active radiation (PAR) and pictograms of local weather conditions, (B) mean temperature of replicate aquaria for each treatment (n = 4), (C) maximum quantum yield of photosystem II (F_V_/F_M_), taken on randomly chosen coral fragments for each species (n = 4) on the three days of the experiment for which s-FTIR data is available, (D) symbiont cell density in randomly selected coral fragments (n = 4) for each species on selected days. Shading and error bars indicate standard error on the mean (n = 4). Asterisks indicate significant difference between control and treatment at *p* < 0.05. Data in blue represent controls and data in red represent heat-treatment.

### Correlation network analyses

Correlations between peak areas within species and cluster were calculated using the “cor” function in R package Weighted Correlation Network Analysis (WGCNA) [39, 40]. Due to the high number of comparisons (*n* = 465 within each group), a conservative approach was adopted by retaining only correlations with *P*-value ≤0.001 and || ≥ 0.3. Undirected graph objects were created from selected correlation data using the graph_from_data_frame function from the R package “igraph” [41] with node size defined by degrees connectivity and edges coloured by direction of correlation. Networks were visualized using the R package “ggraph” with layout = “kk.”

### Identification of differentiating features

The supervised machine learning algorithm Random Forest (RF) was used to detect features in the spectra (based on peak area) that most contributed to their classification in the cluster analysis. Variable correlation was assessed across all data using Spearman’s correlation. One representative variable was selected from each group of highly positively correlated variables (*r_s_* > 0.8), prioritizing variables with known biological function. Random Forest processes were first carried out to obtain the major predictors between each cluster within a given species, and finally between each cluster across all species. Before each analysis, the optimum number (based on Receiver Operating Characteristic, ROC) of mtrys (number of features available at each split) and number of trees were assessed using a grid search across parameters. A training data set based on 60% of the original data with 5-fold resampling cross validation was used for parameter optimization. Due to imbalanced data sets (because of relatively rare cell phenotypes), the training dataset was re-constructed based on re-sampling of the rarer cluster. The final model was run against the remaining validation data (40% of original) and the resulting peak feature importance obtained using the varImp function on the fitted model. To increase prediction stability, the top 50% features identified in the first run were re-run as described above, and the top 5 features presented. See supplementary methods for further details.

### Statistical analyses

All statistical analyses were performed using R statistical software (v4.3.3, [42]). Due to lack of normality and/or homogeneity of variance, non-parametric Wilcoxon Rank Sum was used to assess differences in F_V_/F_M_ and cell density between treatments within days. Nonmetric Multi-Dimensional Scaling (nMDS) was used to assess differences in ITS2 communities within colonies via the function metaMDS (trymax = 100) from the R package “vegan” [43]. Analysis of Similarities (ANOSIM) with bray–curtis dissimilarity matrix and 9999 permutations was used to test for similarity of colonies within species, and SIMPER analysis for pairwise comparisons between species. Generalized Linear Mixed-Effect models (negative binomial) from the R package “lme4” were used to assess the proportion of each cluster (i.e. number of cells with given metabolic profile) within species, treatment and day. To allow for variability across clusters and to take into account repeated measures within colonies, random slopes and random intercept within clusters and colonies, respectively, were included in the model. Time was not included as a crossed effect due to low explanatory power. Linear Mixed-Effect models were used to test for differences in peak area across selected compounds using the function “lmer” in the package “lmerTest” [44]. Cluster was used as a fixed factor and the nested nature of the data accounted for by using a nested random factor structure includingcolony and species. Given the possibility of differing effects within species, species was also included as a random slope. Lastly, time was included as a crossed effect to account for sampling being done over different days. Restricted maximum likelihood was used as logical scalar. Initially, data was tested for normality using the Shapiro–Wilk normality test. If data were not normally distributed, outliers (value > |third quartile +1.5 x inter quartile range|) were removed (0.6%-3% of the data) and data square-root transformed to improve normality.

## Results

### Coral symbiont composition

*Symbiodiniaceae* dinoflagellate communities showed taxonomic consistency across colonies within species (Fig. 1A) and significant differences between coral species (nMDS stress = 0.04; ANOSIM R > 0.99, *P* < 0.0001; Fig. 1A and B). In *A. aspera* the ITS2 sequences were dominated by genome variant A1 (65%-80% of total reads) belonging to a species of *Symbiodinium* (type profile: A1-A1gd-A1ge, Fig. 1C) [45], together with a small proportion of C50b and related sequences from a *Cladocopium*, possibly *C. sodalum* [46]. *A. millepora* was dominated by C50b (23%-77% of total reads) across various ITS2 type profiles (Fig. 1C), but in some cases also harbouring a relatively large fraction of A1 dominated type profiles. This mix of profiles differed significantly from the other two coral taxa which were completely dominated by single genets from the genus *Cladocopium*, with *P. damicornis* dominated by C42a (22%-58% of total reads) and a type profile corresponding to *Cladocopium pacificum* (C42a/C42.2/C1/C1b/C3-C1j-C1au, [47]), whereas ITS2 variant C8 and the related variant C8a dominated colonies of *S. pistillata* (combined 65-77%) with a type profile of an as of yet undescribed species (C8/C8a-C1-C42.2-C42e-C8e-C42ae) (see supplementary Table S1 for cladal differences across species).

### Evidence of heat stress

After two weeks under 50% shading (Fig. 2A) and exposure to increased temperatures (Fig. 2B), we saw a decline in photosynthetic performance (F_V_/F_M_) in all species in the heat treatment by Day 19 (Fig. 2C), with an initial decline starting around Days 15-17 (Supplementary Fig. S1). After an initial increase in cell densities during the first week of the incubation (Supplementary Fig. S2), cell densities started to decline around day 14 compared to control colonies (Fig. 2D), verifying that heat-treated corals were expressing bleaching pathophysiology.

**Figure 3:**
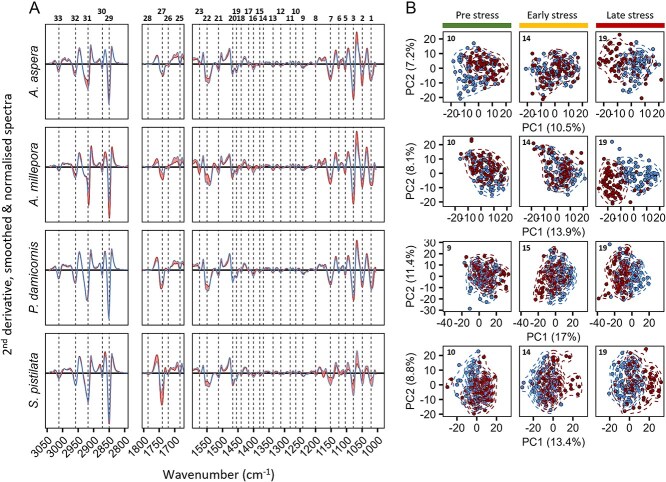
s-FTIR spectral data of individual endosymbionts extracted from four species of corals before and after heat stress. (A) Average spectra of control (blue) and heat-treated cells (red) for each coral species. Shaded area highlight difference in spectra between control and treatment colonies. Vertical stippled lines demark peaks used in further analyses. Numbers at the top are identifiers of each peak for reference (see Table S2). (B) PCA scores plots of whole spectra from individual cells across coral colony and treatment for each key time-point representing pre-stress, early stress and late stress states. Blue: Control cells; red: Heat treated cells.

### Heat stress induces strong shifts in metabolic profiles

A total of 1541 cells were analysed across species for all three time points using s-FTIR microspectroscopy, generating one spectrum per cell. A total of 33 discrete peaks of biological origin within wavenumbers ranging from 3050-1000 cm^−1^ were included in the analyses (Fig. 3A). Spectra averaged by species and treatment on Day 19 (late bleaching) revealed temperature-induced changes in biomolecular composition consistent with previous work [17]; with a downregulation in protein peaks (e.g. #22, amide II) and up-regulation in lipid and carbohydrate peaks (peaks 1-13 and 27, respectively. Figure 3A. See Table S2 for peak assignments). Temporal analysis of spectral profiles using (CA showed increased separation between control and heat-treated profiles along the first principal component (PC1, explaining 10%-17% of the variation) during late stress for all four coral species (Fig. 3B, loadings shown in Supplementary Figs. S4-S7).

### Conserved response diversity within sub-populations of symbiont cells

**Figure 4:**
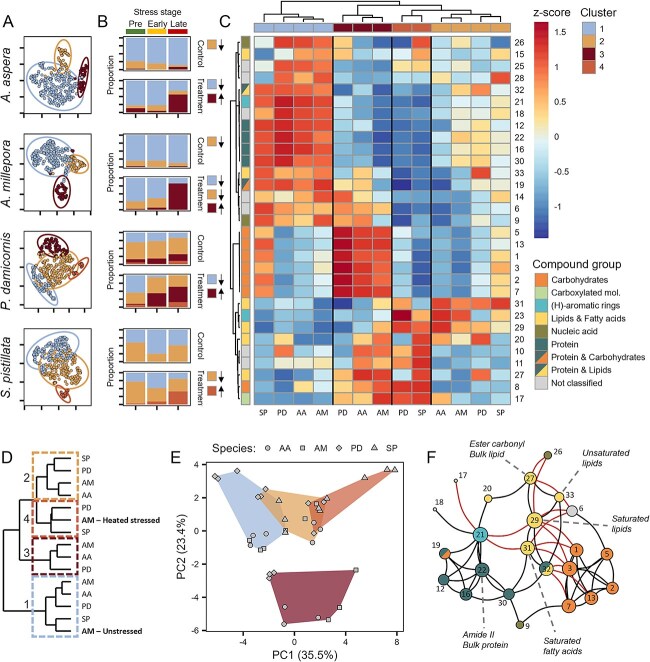
Spectral clustering of s-FTIR spectra of all coral species. (A) Umaps of identified clusters for each species, (B) proportion of cells within each cluster on pre-, early and late stress time points in control (upper panel) and heat-treated (lower panel) colonies of the four coral species. Small boxes with arrows indicate which of the clusters were significantly up (up-arrow) or down (down-arrow) regulated (see fig. S7 and Table S3 for statistics), (C) hierarchical clustering based on z-score similarity of peak areas for each cluster, numbers indicate peak IDs, colours (top) indicate cluster grouping, colours (left side) denote compound grouping, (D) hierarchical clustering of clusters from this study, with the inclusion of data from [17] (bolded AM) showing relative position of control and heat-treated spectral profiles aligning with CL1 and CL4, respectively, (E) PCA of peaks averaged per sample, shape represents species, colours link to clusters, (F) correlation network analysis of peaks across species and treatments. Only correlations that were significant across all species are included, *p* < 0.01, |rho| > 0.3. Black edges indicate positive correlations; red edges indicate negative correlations. Node size indicates centrality, numbers are peak IDs (see Table S2). For clarity, colouring in A and B is based on profile clustering in C.

Clusters showed strong grouping using dimensionality reduction (Fig. 4A) and clear trends in proportional distribution over the selected time points (Fig. 4B). Hierarchical clustering based on z-score similarity resulted in four distinct metabolic profiles (Fig. 4C). These profiles (from now referred to as clusters CL1 to CL4) showed strong similarities across coral species, three of which exhibited clear changes in proportion with temperature stress (CL1, CL3, and CL4) (see Supplementary Fig. S8 and Table S3 for statistics). In the two *Acroporidae* corals (*A. millepora* and *A. aspera*), profile CL1 (blue) dominated in both control and heat-treated coral fragments over time, but was strongly reduced during late bleaching, where CL3 (brown) became dominant (Fig. 4B). In contrast, profile CL2 (yellow) exhibited low prevalence throughout, decreasing in control colonies and in the treatment colonies of *A. millepora*. In *P. damicornis*, a similar pattern was observed for CL1 and CL3, whereas CL2 dominated in both control and heat-treated corals, with little change over time (Fig. 4B). Here, a fourth profile (CL4, orange) was detected that showed no change in prevalence over time. In *S. pistillata*, CL1 and CL2 dominated over time in both treatments, and while CL3 was not observed, CL4 profiles increased with heat stress. The similarity in clustering for the *Acroporidae*, which was distinct from the *Pocilloporidae* (*P. damicornis* and *S. pistillata*), suggests some level of physiological relatedness within the two types of corals.

Comparing the metabolic profiles of the clusters in this study with those from a previous bleaching study on *A. millepora* [17], we found that heat stressed cells (including expelled cells) grouped most closely with CL4 (Fig. 4D) and cells from control treatment correlated with CL1, supporting the assertion that CL4 represents stressed cells, possibly close to expulsion. To verify that the spectral features included in this study adequately describe differences between the metabolic profiles of the coral endosymbionts, we combined the metabolic profiles of colonies and species, and revealed a clear distinction between cluster profiles (Fig. 4E). Conserved responses in biomolecular groups were visualized using correlation network analysis (Fig. 4F). We saw significant positive correlations (*P* < 0.001) within peaks of similar biomolecules (black lines), but negative correlations between the lipid biomarkers and protein or carbohydrate peaks (red lines). Correlations within clusters (across all species), showed no clear connection between carbohydrates and other biomolecules in CL1, contrasting with all other clusters that exhibited negative correlations between saturated-lipids and -fatty acids and carbohydrates (Supplementary Fig. S9). While only correlative, these relationships suggest a tradeoff between lipid accumulation and other processes, indicative of physiological stress [48].

### Protein and carbon storage are important indicators of bleaching stress in coral symbionts

**Figure 5:**
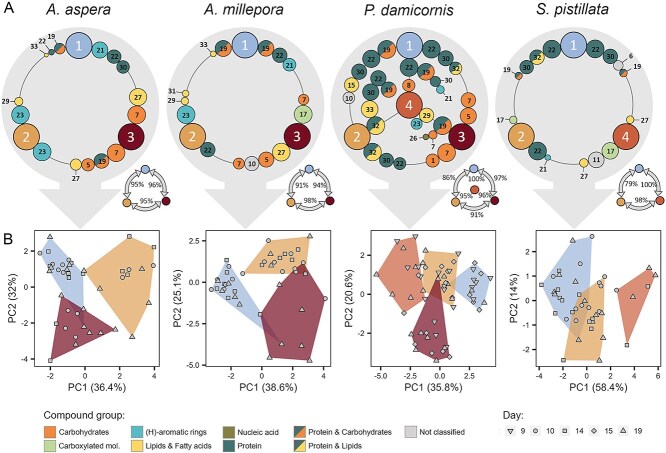
Key discriminating peaks for metabolic profiles within species of corals. (A) Top 5 peaks from random Forest (RF) that differentiate respective clusters within a given species. Large, numbered circles indicate cluster (1-4), small, coloured circles between two clusters contain IDs of peaks and colours indicate compound annotation. Peaks ordered and sized according to importance where circles closer to the cluster are more important for differentiating clusters. Percentages indicate prediction accuracy. (B) Principal component analysis (PCA) based on top 5 differentiating peaks as per RF analysis (above) within each coral sample across time (day) and treatment. Convex hull shading connects points relating to a specific cluster: CL1 (blue), CL2 (yellow), CL3 (brown), CL4 (orange). Shapes indicate day of incubation.

By using the RF algorithm, we determined the strongest predictors for each of the four metabolic profiles within and across all species (Fig. 5). Generally, each profile could be predicted with high accuracy (>90%) against any of the other profiles, even when including only the top 50% most important features (Fig. 5A). Despite considerable variability in the top five specific feature importances between species, some clear patterns emerged: Prediction of profile CL1 against other profiles was dominated by protein related features, whereas prediction of CL3 relied strongly on carbohydrate related features. In most cases protein was also important in differentiating profile CL2 from CL3 and CL4, supporting the indication of higher protein in these cells (Fig. 5A). Principal component analysis of the top five features for each cluster within each species were able to separate profiles across days (Fig. 5B), with 35%-58% of total variance explained by PC1 and 14%-32% explained by PC2.

**Figure 6:**
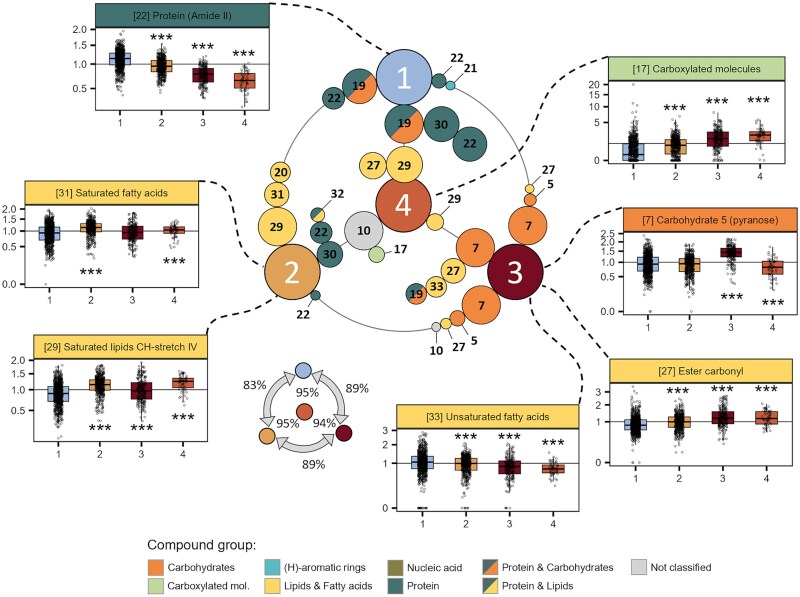
Major discriminating peaks for metabolic profiles found in all four coral species and relative concentration of key peaks for each cluster. (CENTRE) top 5 discriminating peaks for each cluster across all species as identified using RF analysis. Peaks ordered and sized according to importance. Percentages indicate prediction accuracy. Box and whisker plots of peak areas (sqrt root transformed) for each chemically known peak identified across clusters (numbered 1-4). Number and colour of label refer to peak ID and compound annotation, respectively. Asterisk under cluster indicates significantly different from CL1 using linear mixed effect model at ^***^ p < 0.001 (see Table S4 for statistics).

Combining all species, Random Forest analysis was able to predict profiles with an accuracy of 83%-95% (Fig. 6), verifying similar features were discriminating individual profiles across the different corals (since initial clustering was performed *within* species, this was not a given). Here, the conserved pattern across species becomes clearer, with CL1 defined by higher protein than all other clusters, CL2 having higher protein than CL3 and CL4 but higher lipid, specifically saturated lipids and fatty acids, than CL1. Profile CL3 was defined by higher carbohydrate levels and CL4 by high lipid and higher levels of carboxylated molecules (Fig. 6; Table S3).

## Discussion

Here we have shown that multiple, metabolically distinct symbiont cells can exist at the same time within a coral colony, with similar biomolecular characteristics found across four coral species harbouring different symbiont type profiles (ITS2). These data reveal that metabolic responses of the symbionts to environmental conditions carry strong similarities across evolutionarily divergent species of coral. Furthermore, we have shown that symbiont metabolic characteristics in the coral change predictably under conditions of temperature stress. These complementary metabolic shifts suggest conserved changes in the endosymbiont metabolism and the strong similarities in metabolic fingerprints across divergent ITS2 profiles highlights a general trend rather than strain specific outcomes. While our data suggest a strong metabolic commonality under similar environmental conditions across corals, we observed differences in the proportion of different metabolic profiles between the *Acroporidae* and the *Pocilloporidae* corals, possibly reflecting differences in coral host regulation in response to endosymbiont physiology.

Based on the proportional contribution to the total population over time and the biomolecular composition of each profile (Fig. 4A and B), we conclude that profile CL1 represents non-stressed cells—with the highest protein and nucleic acid content and relatively lower carbon-storage content (lipid and carbohydrate). Our interpretation is supported by the grouping of CL1 with control (non-heat treated) endosymbiont cells from a previous study [17], and low carbon storage has previously been linked to a healthy symbiosis, reflecting continuous transfer of photosynthetic products to the host [49–51].

**Figure 7:**
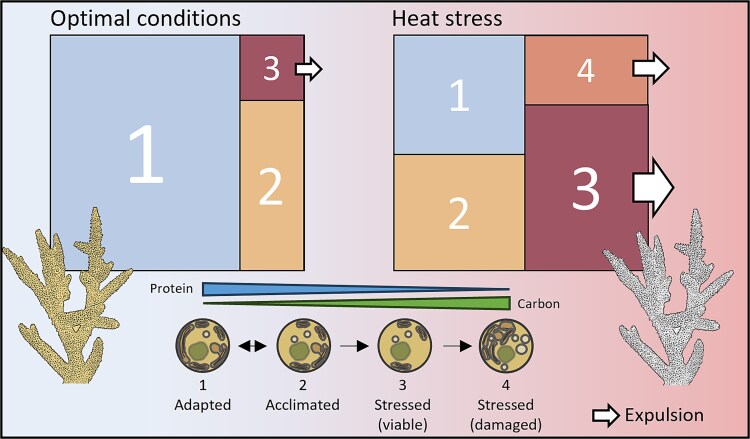
Conceptual overview of the endosymbiont community dynamics within coral colonies. Under optimal conditions (left), the coral algal phenome is dominated by healthy algae (1 and 2) with high protein content, mainly from photosystem components, and low carbon content due to efficient transfer to the coral host. The two healthy profiles represent cells adapted to the long-term environmental conditions (1) versus endosymbionts acclimated to short term changes (2), and endosymbionts may be able to transition between the two states as conditions fluctuate. As physiological stress incurs (right), the metabolic profiles of individual alga shifts depending on their individual state of health, resulting in changing proportions across the coral tissue. As the stress levels increase, the coral becomes dominated by endosymbionts with low protein content due to photosystem reduction and increased carbon content from reduced transfer to the host (3 and 4). While some of these cells may still be photosynthetically competent (3) and likely to survive post-expulsion, others (4) may be in the process of degradation and likely not to survive. Given the compromised metabolic states, cells with profiles 3 and 4 may be non-symbiotic and therefore in the process of expulsion from the coral (white arrows).

The increase in the proportion of CL3 and CL4 profiles with heat treatment over time strongly advocates that these cells represent a stressed state. Both profiles were characterized by increased lipids and/or carbohydrates and lower protein levels, corroborating previous work showing a reduction in protein content, and an accumulation of lipid in stressed and expelled symbionts [17, 20, 51]. Additionally, both profiles exhibited the highest relative increases in carboxylated molecules, likely driven by accumulation of free amino acids from protein degradation. These heat-induced metabolic shifts reflect strong alterations in host–symbiont carbon and nitrogen regulation, which is important for sustaining healthy symbioses [23, 24, 50]. Recently, a study by Cui et al. (2023) demonstrated the importance of nitrogen cycling for sustaining the holobiont metabolism, by looking at nutrient transport between host and symbiont. They found that during symbiosis, increased glucose, or algal presence induced up-regulation of glucose and amino acid transporters. Our results contribute new experimental evidence for the importance of metabolic interactions between the host and symbiont in regulating the symbiosis.

The greatest metabolic distinction between the two stressed phenotypes (CL3 and CL4), is the marked accumulation of carbohydrates in CL3. This suggests that CL3 cells may yet have been photosynthetically competent, and the accumulation of carbon likely resulted from a breakdown in the transfer to the host cell. This interpretation is congruent with a study looking at carbon and nitrogen assimilation in *Cladocopium* spp. and host coral tissue, which showed that under heat stress, symbiont assimilation continued unimpeded, but there was a significant decline in host assimilation, demonstrating breakdown of nutrient translocation (Kemp et al 2023).

The metabolic profile of CL4 shared strong similarities with the average metabolic profile of heat-treated cells from *A. millepora* [17], supporting the assertion that this cluster represents stressed cells. The lack of carbohydrate accumulation in CL4 cells may have resulted from complete degradation of the photosynthetic machinery as observed previously in a fraction of heat stressed endosymbionts of *A. millepora* [17].As such, the CL4 profile may represent severely compromised or dying cells. Interestingly, while the CL4 profile was detected in both pocilloporid corals it was not detected in either of the Acroporidae in this study. This could be a result of timing combined with differences in expulsion management between the two types of corals [52], whereby the cells matching the CL4 profile were already expelled from the *Acroporid* corals at the time of final sampling.

Of the four metabolic profiles detected in this study, CL2 was the only one to change in proportion under control conditions, with a decline in both Acroporid species. In combination with the marked difference in relative proportions of CL1 and CL2 profiles between the Acroporid and Pocilloporid corals, these data suggest a potential difference in the role of this profile within the two coral groups. The CL2 profile is characterized by high levels of saturated lipid and fatty acids (peaks 29 & 31) compared to other profiles, a trait typically associated with adaptation to increased temperatures via decreasing membrane fluidity. Indeed, in a recent study, saturated lipids and fatty acids were found to be the strongest metabolome features in non-bleaching over bleaching coral phenotypes [19]. Equally, an increase in saturated fatty acids may also result from heightened biosynthesis of de-novo fatty acids during cell differentiation or metabolic adjustments, which is typical of healthy cells adjusting to new conditions. Cells with the CL2 profile were also distinctive in having a higher level of protein and overall lower lipid content (other than saturated lipids) compared to profiles CL3 and CL4. Lastly, both pocilloporids, which contained a large proportion of CL2 cells, exhibited high maximum quantum yield (F_V_F_M_) throughout the incubation period until the late heat stress stage (Supplementary Fig. S1), suggesting that the dominant CL2 cells were still photosynthetically competent. Taken together, these data indicate that cells with a CL2 profile were metabolically proficient and may represent a more heat resistant or thermally acclimated endosymbiont phenotype compared to CL1 cells. While CL2 cells did not accumulate over time in heat treated colonies, as might be expected from more resilient phenotypes, they dominated the phenomes of control colonies in the two Pocilloporid corals, and were more abundant than CL1 cells at the end of the heat treatment, which may come about from preferential retention in the coral tissue.

The results from this study highlight that heat-stress is a pathophysiological response that is initiated within individual cells, resulting in a shifting continuum of metabolic states from healthy to severely stressed throughout the coral tissue (Fig. 7). The presence of multiple distinct physiological states of coral algal symbionts during stress demonstrates how the physiological cause of the degeneration of the symbiosis may be obscured in bulk analyses: Initially, affected cells are relatively rare, thus escaping detection on a background of healthy cells. Later the coral tissue may be dominated by more severely compromised cells that exhibit stress indicators that are a consequence of but not necessarily directly relevant to the breakdown of the symbiosis. Here, to escape the fog of averaged signals, we analysed the metabolomes of individual endosymbiotic cells and detected multiple metabolic profiles of algal endosymbionts across time and treatment. Each profile exhibited distinct characteristics, both with respect to occurrence over time and metabolic features, which may provide clues to their meaning. While the interpretation of the different profiles requires further validation, these data have revealed, for the first time, hitherto unknown metabolic sub-populations within individual coral colonies that respond dynamically to heat stress conditions, with similar effects across multiple coral species and a distinct, cascade like transition from high protein/low carbon cells to low protein/high carbon cells (Fig. 7).

## Supplementary Material

Nielsen_et_al-supplementary_ycag099

## Data and materials availability

All data are available in supplementary files.
