# Supplementary material for: Heat stress reproportions distinct metabolic sub-populations of coral-algal endosymbionts
Source: ISME Commun. 2026 Apr 20;6(1):ycag099. doi: 10.1093/ismeco/ycag099 (PMC13155119; doi:10.1093/ismeco/ycag099)
Supplement: Nielsen_et_al-supplementary_ycag099 [file nielsen_et_al-supplementary_ycag099.pdf]

Heat stress reproportions distinct metabolic sub-populations of coral-algal endosymbionts

Supplementary information

Daniel A. Nielsen[1*], Philip Heraud[2], Trent D. Haydon[3], Katherina Petrou[1]

[1]School of Life Sciences, University of Technology Sydney, Ultimo, New South Wales, Australia.
[2]School of Chemistry, Monash University, Clayton, Victoria, Australia
[3]Center for Genomics and Systems Biology, New York University Abu Dhabi, PO Box 129188, Abu Dhabi, UAE

*Author for correspondence: Daniel A. Nielsen

Email: daniel.nielsen@uts.edu.au

Detailed methods

Endosymbiont extraction

For single-cell investigation of biomolecular composition, intact endosymbiotic algal cells were extracted from each coral colony by placing a coral fragment in a 50 mL centrifuge tube containing 5 mL of 0.22 μm filtered seawater and firmly hitting the base of the tube against a hard surface (Nielsen et al 2018). This extraction procedure resulted in a cell slurry with a high percentage of intact endosymbiotic host cells (see Supplementary Figure S3). Once extracted, cells were gently spun down (500 rpm), resulting in precipitation of the heavier symbiont containing cells. The supernatant with coral mucus and less dense host tissue was removed and the remaining cells subsequently re-suspended in 100 μL FSW and preserved in 2% formalin for analysis on the Infrared Microspectroscopy (IRM) beamline at the Australian Synchrotron (ANSTO, Melbourne).

Symbiont classification via ITS2 sequencing and analysis

DNA was extracted from isolated symbionts at T0 (see Symbiont cell density) using DNeasy Blood and Tissue kit (QIAGEN, Hilden, Germany) according to manufacturer's specifications. To assess the Symbiodiniaceae community composition, extracted DNA was amplified via PCR reactions, consisting of 1.5μl of ITSintfor2 and ITS2-reverse primers [1, 2] with attached Illumina adaptors, 12.5μl of Q5 Hot Start High-Fidelity 2x Master Mix (New England Biolabs, Ipswich, MA, USA) and diluted DNA (20-30ng l^{-1}) for a total 25 μl reaction. PCR cycling conditions were as follows: initial denaturation at 98°C for 30s, followed by 25 cycles of 98°C for 10s, 55°C for 15s, and 72°C for 30s, with a final extension at 72°C for 2 min. Amplicons were prepared for sequencing following Illumina's standard protocol for library preparation. Purification was performed using calibrated AMPure XP beads (Beckman Coulter, USA) after

which the DNA was indexed with the Nextera XT kit (Illumina USA) under standard PCR conditions. Indexed amplicons were pooled and sequenced on the Illumina MiSeq platform (2x 300bp) at the Australian Genomic Research Facility (Victoria, Australia). Resulting FASTQ files were then processed using the SymPortal analytical framework, where paired-ended sequences were quality controlled using mothur 1.43.0 [3], the blast+ suite of executables [4], and minimum entropy decomposition[5]. Following quality control, intragenomic ITS sequence variants representing different Symbiodiniaceae genotypes were used to plot ITS2 sequence counts of each coral sample.

Synchrotron-based FTIR microspectroscopy

Synchrotron FTIR microspectroscopy was performed on three sets of samples for each species at the Australian Synchrotron IRM beamline. Due to the high temporal resolution of sampling, samples could be obtained from each species that represented similar stages in the bleaching process (based on cell counts), independent of differences in timing resulting from difference in physiologies. As such, samples were chosen for analysis from each species representing pre-bleaching (day 9-10), early-bleaching (days 14-15) and late bleaching (day 19). Formalin fixed *in-hospite* cells were loaded into a micro compression cell (modified compression cell, Spectratech inc., Oak Ridge, USA; [6]) with 1 mm thick, IR transparent, CaF_2 window. Measurements were made on hydrated cells, a method shown to limit resonant mie scattering (RmieS) artefacts [7]. Each endosymbiotic algal cell was visually identified through the microscope at 400 times magnification (Supplementary Figure S3). Positive identification and selection were based on the presence of at least one (but often more) symbiont encased in a host cell membrane, with no surrounding tissue that could interfere with the transmission signal. Seeing as the focus of this study was on the physiology of the endosymbiotic algae

specifically, presence of a host membrane was essential to verify that the algae was indeed endosymbiotic at the time of sampling, as opposed to just residing within the coral host gastrovascular cavity. For this reason, no effort was made to remove the host membrane around the algal cell during pre-processing, and any algal cells without a host membrane were excluded from the study. This process ensured that only endosymbiotic cells were included for analysis. While the presence of the host cell around the symbiotic algal cell invariably has some effect on the absorbance spectra, this effect is negligible compared to the absorbance of the algal cell due to the thinness (~1µm) and very low absorbance of the host membrane and content (see [8] for further information). Spectra were acquired over the measurement range 4000-800 cm^{-1} with a Vertex 80v Fourier Transform Infra-Red (FTIR) spectrometer (Bruker Optics, Ettlingen, Germany) in conjunction with an IR microscope (Hyperion 3000, Bruker) fitted with a liquid nitrogen cooled mercury cadmium telluride detector. The microscope was connected to a computer-controlled microscope stage contained within a specially designed box purged with dehumidified air. Co-added interferogram scans (n=32-64) were collected at a 4 cm^{-1} wavenumber resolution. Spectral measurements were made on double endosymbiotic cells (host cell with two algal endosymbionts) using transmission mode with an aperture size of 4 µm x 4 µm. Typical scans comprise one spectrum per algal endosymbiont. Spectral acquisition and instrument control were performed using Opus 6.5 software (Bruker).

Classification of cell types: Cluster analysis

To detect spectra representing cells in different metabolic states, spectral clustering was employed using the Spectrum function in the R package Spectrum [9]. Spectral clustering is a non-parametric clustering method that uses heuristics based on graphs and connectivity, and can identify clusters of arbitrary shapes and is robust to noise and outliers [10]. To reduce the amount of redundant and noisy data that can adversely affect the clustering algorithm, all positive values (none-informative) were set to zero and the remaining data points converted to

positive values for ease of interpretation. Data from all days and treatments were analysed together for each species to ensure that the underlying assumptions for cluster generation were identical across all samples. Thus, the following steps were performed on the data for each species separately: all zero variance wavenumbers across samples were excluded from the spectra; additionally, low variance wavenumbers, which are not suitable for scaling, were identified using the function nearZeroVar from the R package "caret" [11] and subsequently excluded. This procedure reduced total wavenumbers in each spectrum used in the analysis by 32-44% depending on the species. To further reduce noise the data were condensed into principal components using the base R function "prcomp" with scaling set to "TRUE". The number of principal components to be included for each clustering was first evaluated using the function findPC [12] including 12-72 PCs and mean aggregation. The final number of PCs were selected by testing the clustering efficiency of different numbers of PCs around the suggested value, resulting in the selection of 6-12 PCs depending on species (AA: 9PCs, AM: 6PCs, PD: 12PCs, SP: 7PCs). Spectral clustering was first performed without pre-specifying the number of clusters, allowing the most appropriate number of clusters to be determined by the algorithm. This resulted in the selection of four and three clusters for PD and SP, respectively, however for AA and AM the algorithm output resulted in over-fitting of the data. Therefore, for these species a heuristic approach was employed, running the algorithm with a set number of clusters (from 2 to 15) and evaluating the result based on minimum overlap of clusters when projected into a Umap space using the R package "umap" [13]. From this approach, it was considered that three clusters were appropriate for both AA and AM, and the clustering algorithm was re-run using the option for fixed number of clusters (fixk = 3).

Identification of differentiating features

The supervised machine learning algorithm Random Forest (RF) was used to detect features in the spectra (based on peak area) that most contributed to their classification in the cluster

analysis. Due to the nature of vibrational spectroscopy, many peaks relate to similar macromolecules (i.e. carbohydrates, lipid or proteins) and while peaks may relate to different aspects of the macromolecule, they are often strongly correlated due to quantitative relationships. Therefore, variable correlation was assessed across all data using Spearman's correlation analysis from the "cor" function in R package WGCNA [14, 15]. To reduce overfitting in the RF analysis, one representative variable was selected from each group of highly positively correlated variables ($r_s > 0.8$), prioritising variables with known biological function. This process resulted in the removal of 5 peaks (IDs 2, 3, 12, 13, 16; Table S2). The remaining variables were subsequently used in the RF analysis using the train function (method = rf) in the "caret" package, with cluster group as the dependent variable. Random Forest processes were first carried out to obtain the major predictors between each cluster within a given species, and finally between each cluster across all species. Before each analysis, the optimum number (based on Receiver Operating Characteristic, ROC) of mtrys (number of features available at each split) and number of trees were assessed using a grid search across mtrys 1:10 and between 200 and 2000 trees with a step size of 200. Node-size was set to 1 to allow for highest tree resolution and the value for max-nodes as per the standard function settings. The model was trained on a training data set based on 60% of the original data with 5-fold resampling cross validation for parameter optimisation. Due to highly imbalanced data sets (because of relatively rare cell phenotypes), the training dataset was re-constructed based on random re-sampling of the rarer cluster up to three times the original population size, while restricting the more common cluster to the same final population size. The final model was run against the remaining validation data (40% of original) and the resulting peak feature importance obtained using the varImp function on the fitted model. To increase prediction stability, the top 50% features identified in the first run were re-run as described above, and the top 5 features presented.

Tables and figures

Table S1. Output of SIMPER analysis for symbiont community composition. Pairwise comparison of ITS2 diversity across coral species.

Am & Aa (ITS2)	Av. Abund. (*Am*)	Av. Abund. (*Aa*)	Av. Diss. %	Cum. %	*p*	Significance
A1	0.12	0.71	31	38	5.00E-04	***
C50b	0.41	0.028	19	62	1.00E-04	***
A1gd	0.038	0.14	6.4	70	0.002	**
C3	0.12	0.0077	5.5	77	1.00E-04	***
C50p	0.11	0.0083	5.2	84	0.0028	**
A1ge	0	0.074	3.8	88	1.00E-04	***
C8	0	8.20E-05	0.0042	100	0.9943	*
Am & Pd (ITS2)	Av. Abund. (*Am*)	Av. Abund. (*Pd*)	Av. Diss. %	Cum. %	*p*	Significance
C42a	0	0.41	21	22	1e-04	***
C50b	0.41	0	21	43	1e-04	***
C1	0	0.14	7.4	50	1e-04	***
C42.2	0	0.13	6.8	57	1e-04	***
A1	0.12	5.7e-06	6	63	0.9909	
C50p	0.11	0	5.4	69	6e-04	***
C3	0.12	0.019	5	74	1e-04	***
C1b	0	0.06	3.1	82	1e-04	***
A1gd	0.038	0	1.9	86	0.9426	
Am & Sp (ITS2)	Av. Abund. (*Am*)	Av. Abund. (*Sp*)	Av. Diss. %	Cum. %	*p*	Significance
C8	0	0.47	24	24	1e-04	***
C50b	0.41	0	21	45	1e-04	***
C8a	0	0.24	12	58	1e-04	***
A1	0.12	1.5e-05	6.1	64	0.9938	
C3	0.12	0.0041	5.8	70	1e-04	***
C50p	0.11	0	5.5	75	3e-04	***
C42.2	0	0.048	2.5	78	0.8452	
C1	0	0.048	2.5	80	0.9079	
A1gd	0.038	5.5e-06	2	84	0.9427	
A1ge	0	7.6e-06	0.0004	100	0.9948	
Aa & Pd (ITS2)	Av. Abund. (*Aa*)	Av. Abund. (*Pd*)	Av. Diss. %	Cum. %	*p*	Significance
A1	0.71	5.7e-06	36	36	1e-04	***
C42a	0	0.41	21	57	1e-04	***
C1	0	0.14	7.3	65	1e-04	***
A1gd	0.14	0	7.2	72	3e-04	***
C42.2	0	0.13	6.7	79	1e-04	***
A1ge	0.074	0	3.7	87	1e-04	***
C1b	0	0.06	3	90	1e-04	***

C50b	0.028	0	1.4	93	0.9923	
C3	0.0077	0.019	0.59	95	0.9924	
C50p	0.0083	0	0.42	97	0.9578	
C8	8.2e-05	0	0.0041	100	0.9972	
Aa & Sp (ITS2)	Av. Abund. (*Aa*)	Av. Abund. (*Sp*)	Av. Diss. %	Cum. %	*p*	Significance
A1	0.71	1.5e-05	36	37	1e-04	***
C8	8.2e-05	0.47	24	60	2e-04	***
C8a	0	0.24	12	73	2e-04	***
A1gd	0.14	5.5e-06	7.3	80	3e-04	***
A1ge	0.074	7.6e-06	3.8	84	1e-04	***
C42.2	0	0.048	2.5	86	0.861	
C1	0	0.048	2.4	89	0.9158	
C50b	0.028	0	1.4	90	0.9944	
C50p	0.0083	0	0.42	96	0.96	
C3	0.0077	0.0041	0.31	97	1	
Pd & Sp (ITS2)	Av. Abund. (*Pd*)	Av. Abund. (*Sp*)	Av. Diss. %	Cum. %	*p*	Significance
C8	0	0.47	24	27	1e-04	***
C42a	0.41	0	21	50	1e-04	***
C8a	0	0.24	12	64	1e-04	***
C1	0.14	0.048	4.9	69	0.005	**
C42.2	0.13	0.048	4.3	79	0.0141	*
C1b	0.06	0	3.1	82	1e-04	***
C3	0.019	0.0041	0.74	90	0.9918	
A1	5.7e-06	1.5e-05	0.00092	100	1	
A1ge	0	7.6e-06	0.00039	100	0.997	
A1gd	0	5.5e-06	0.00028	100	1	

Table S2: **Infra-red band assignments of selected spectral peaks.** "ID" is the numeric reference value for the specific peak. "Peak" is the wavenumber at maximum absorbance for given peak. "Frequency range" is the wavenumber range of the peak. "Band assignment" is the expected molecular origin of the vibrational pattern giving rise to the peak. "Biological compound group" is the type of molecule that is likely to mostly contribute to the specific vibrational pattern. "Not classified" means we were unable to assign an origin of the respective peak.

ID	Peak (cm^{-1})	Frequency range (cm^{-1})	Band assignment	Biological compound group	Reference
1	1021	1035 - 1010	ν_s (C-O): Carbohydrate	Carbohydrate	[16]
2	1049	1060 - 1038	ν_s (C-O): Carbohydrate	Carbohydrate	[16]
3	1078	1090 - 1070	ν_s (C-O): Carbohydrate	Carbohydrate	[16]
5	1104	1113 - 1099	ν_s (C-O): Carbohydrate	Carbohydrate	[16]
6	1125	1133 - 1117	-	Not classified	-
7	1152	1166 - 1140	ν_s (C-O): Carbohydrate	Carbohydrate	[16]
8	1200	1214 - 1190	ν_s (C-O): Carbohydrate	Carbohydrate	[16]
9	1241	1252 - 1232	ν_{as} (PO_2^-): Nucleic acid	Nucleic acid	[17]
10	1264	1270 - 1256	-	Not classified	-
11	1282	1291 - 1277	-	Not classified	-

12	1312	1325 - 1300	Amide III β turns	Protein	[18]
13	1337	1346 - 1328	δ (CH) / δ (OH): Carbohydrate	Carbohydrate	[16]
14	1368	1375 - 1363	-	Not classified	-
15	1380	1390 - 1375	δ_s (CH_2, CH_3)	Lipid	[19-21]
16	1400	1410 - 1390	ν_s(COO^-) associated with δ_s(CH_3) of proteins	Protein	[22, 23]
17	1416	1425 - 1411	ν_s (COO^-): Carboxylated molecules	Carboxylated molecules	[19]
18	1440	1445 - 1434	-	Not classified	-
19	1455	1460 - 1445	δ_{as} (CH_2, CH_3)	Protein & Carbohydrate	[19-21]
20	1467	1477 - 1461	δ_{as} (CH_2, CH_3)	Lipid	[19-21]
21	1513	1522 - 1505	ν(C=C) Semi-circle stretch	Aromatic and heteroaromatic rings	[24, 25]
22	1550	1560 - 1530	Amide II	Protein	[26, 27]
23	1574	1583 - 1569	ν(C=C) Quadrant stretch	Aromatic and Heteroaromatic rings	[24, 25]
25	1683	1688 - 1676	-	Not classified	-
26	1721	1725 - 1711	ν_s (C=O): Nucleic acid base pairs	Nucleic acid	[17]
27	1740	1750 - 1731	ν_s (C=O): Ester carbonyl	Lipid	[28-30]
28	1786	1795 - 1777	-	Not classified	-
29	2850	2860 - 2842	ν_s (CH_2)	Saturated Lipids	[28-30]
30	2872	2879 - 2868	νs (CH_3)	Protein & Saturated Lipids	[28-30]
31	2918	2938 - 2910	νas (CH_2)	Saturated Lipids	[28-30]
32	2958	2972 - 2950	νas (CH_3)	Protein & Saturated Lipid	[28-30]
33	3012	3025 - 3000	ν(CH) of *cis* C=CH–	Unsaturated Lipid	[28-30]

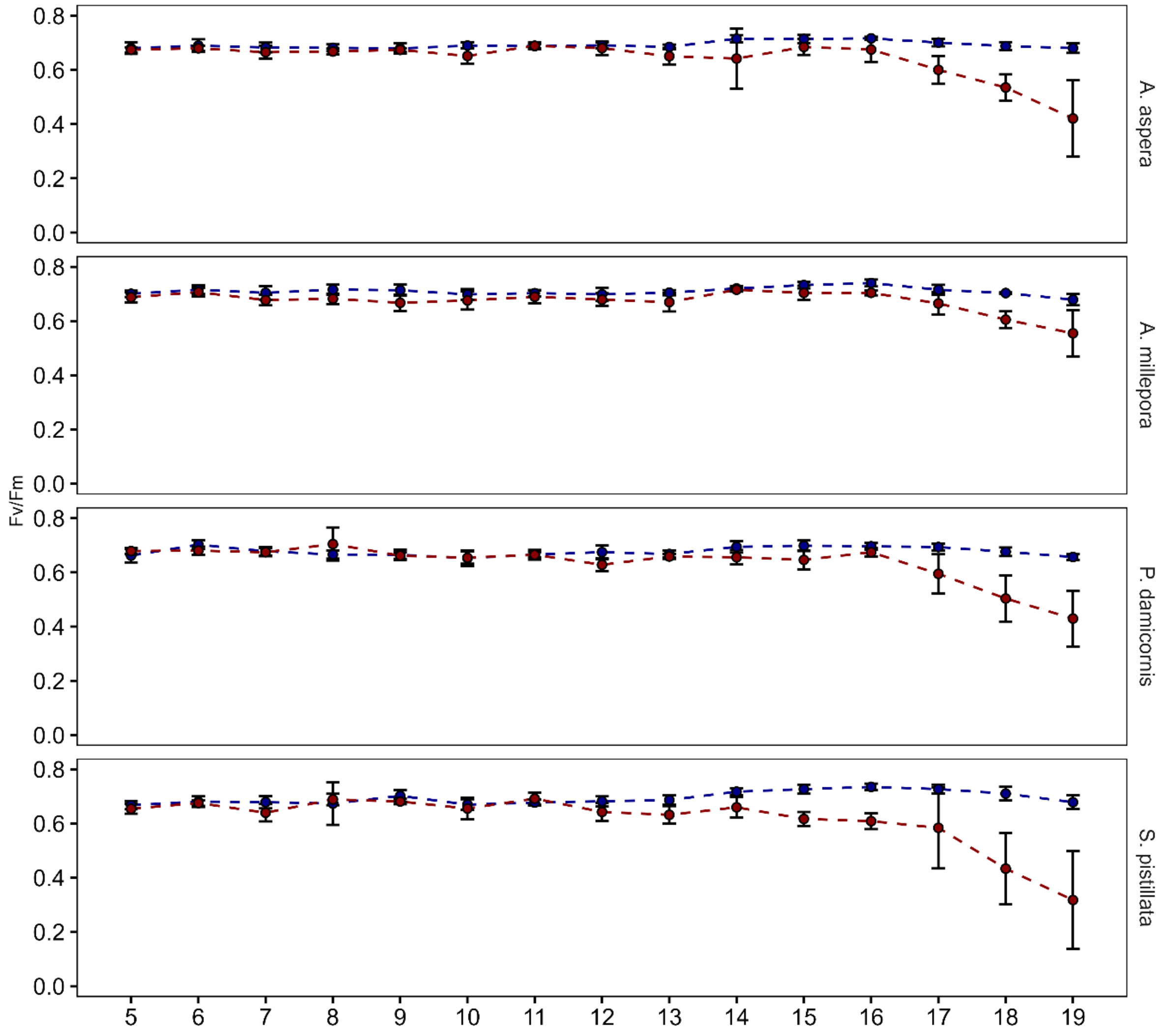

Figure S1. Mean maximum quantum yield of PSII of coral fragments. Random fragments were measured on each day of the incubation period. Errorbars indicate standard error (n = 4)

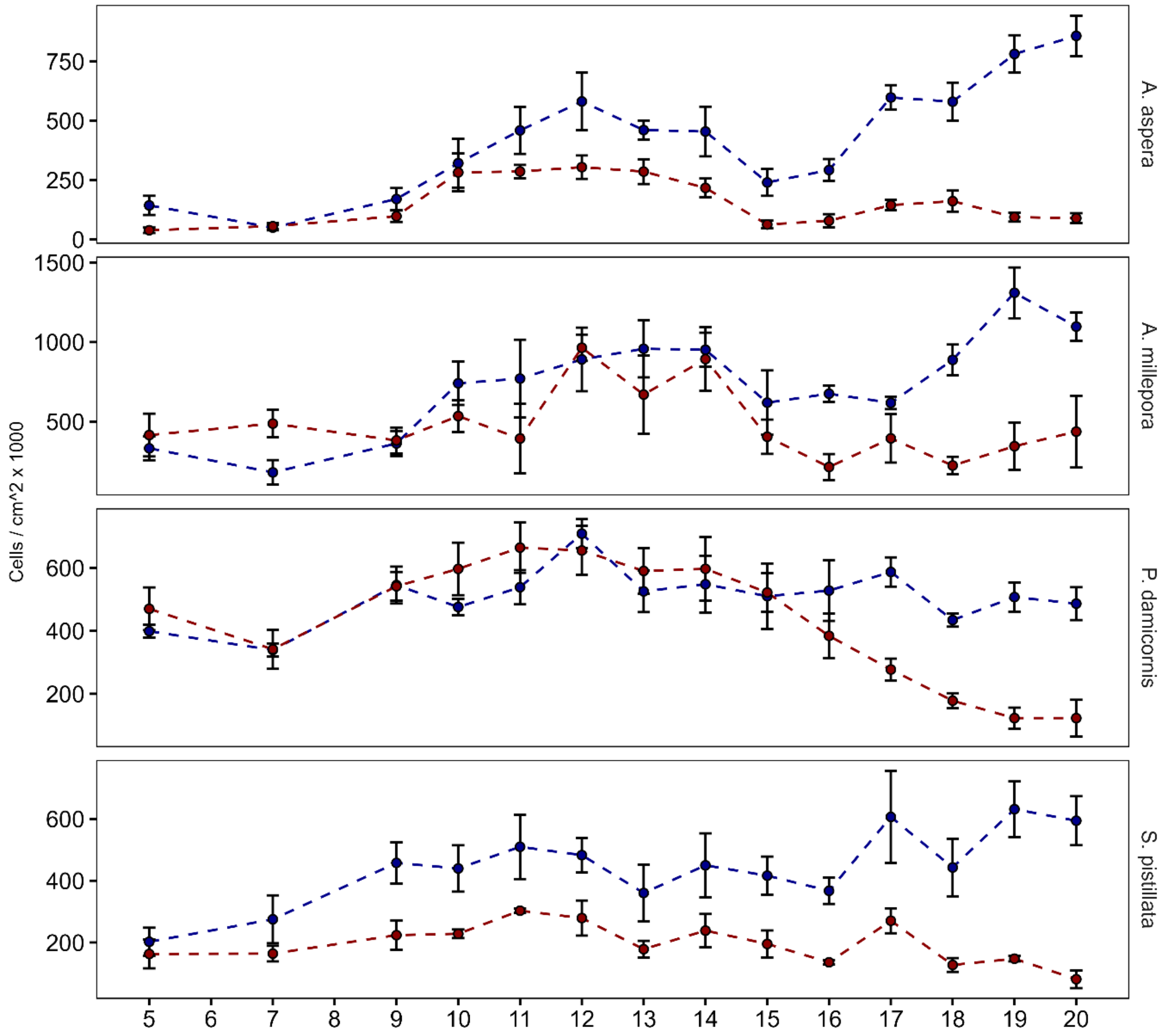

Figure S2. Mean tissue endosymbiont cell densities across species and coral colonies on each day of the incubation period. Increases in cell density were apparent at the beginning of the experiment, which was likley due to coral colonies experiencing some level of bleaching on the reef flat prior to collection and the more emeanable conditions during the initial stages of the experiment. Errorbars indicate standard error (n=4).

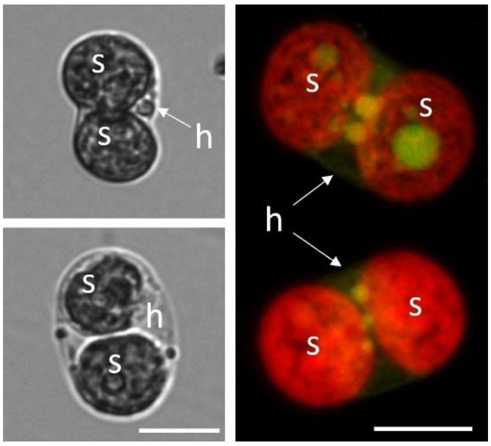

Figure S3. Example microscopy images of coral host cells with two endosymbiotic algae. Left, light microscopy images, right) fluorescence images with host lipid bodies and membranes stained with a fluorescent dye (Image-IT™, BODIPY 581/591 C11) (green/orange) and algal autofluorescence (red). “h”: host membranes, “s”: algal symbiont. Scale bars are 10 ɥm.

A. aspera [Day 19]

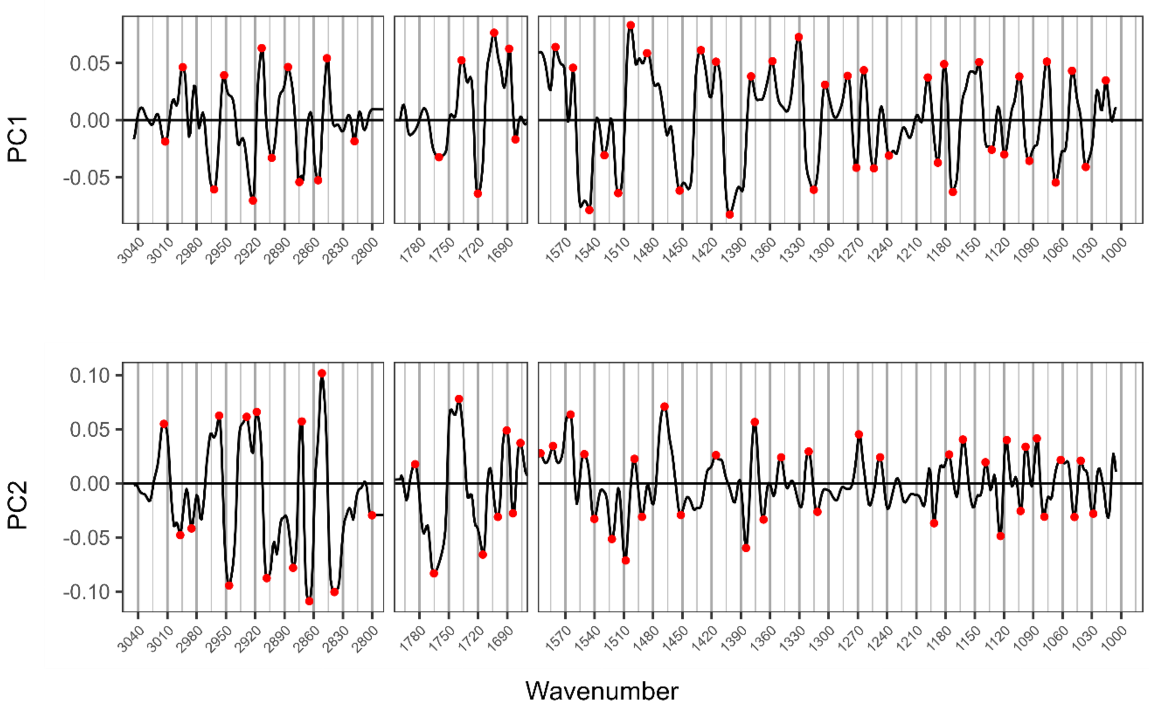

Figure S4. PCA loadings of *Acropora aspera* on day 19. Showing the contributions of each biomolecule to PC1 and PC2 variation, where negatively loaded bands have the strongest absorbance in positively scored samples and positively loaded bands will have the strongest absorbance in negatively scored samples.

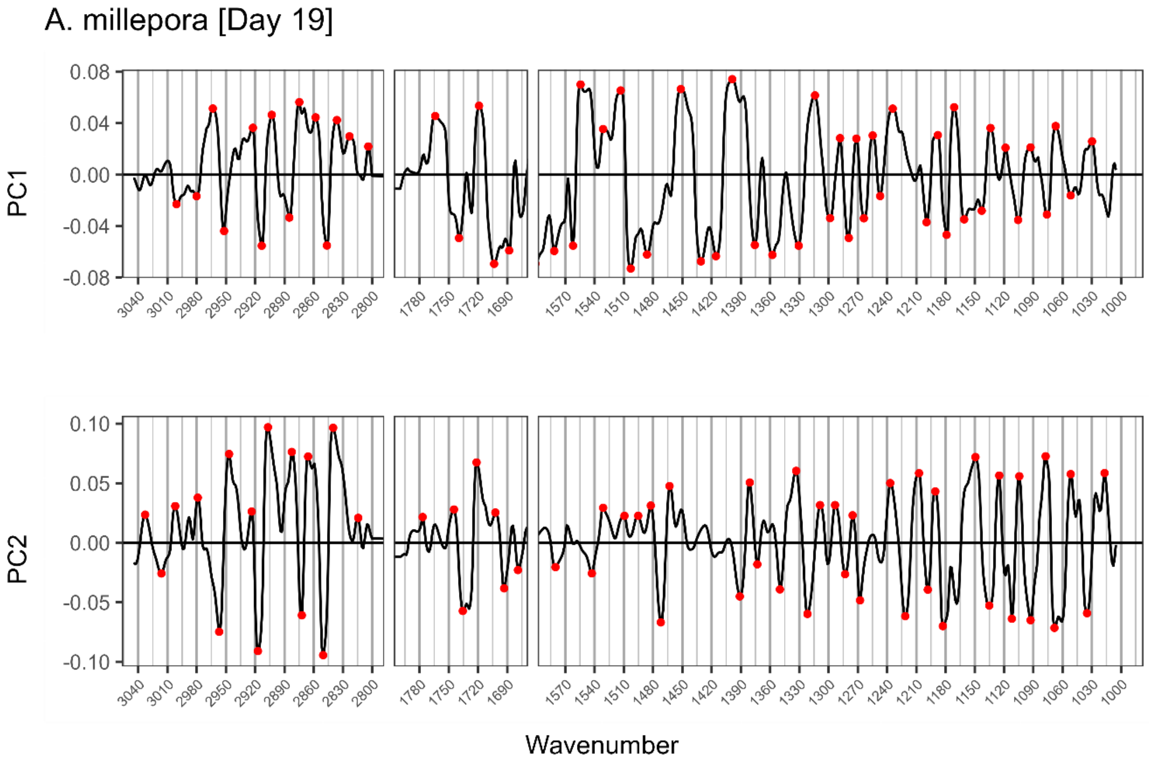

Figure S5. PCA loadings of *Acropora millepora* on day 19. Showing the contributions of each biomolecule to PC1 and PC2 variation, where negatively loaded bands have the strongest absorbance in positively scored samples and positively loaded bands will have the strongest absorbance in negatively scored samples.

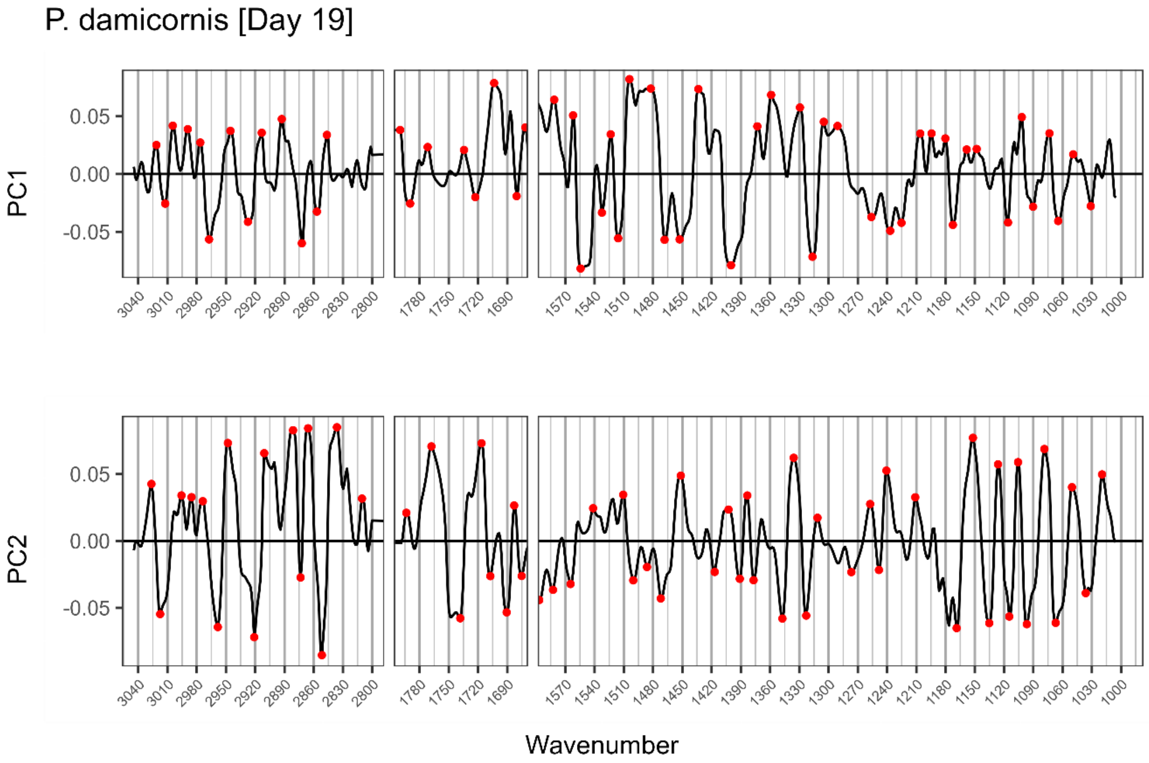

Figure S6. PCA loadings of *Pocillopora damicornis* on day 19. Showing the contributions of each biomolecule to PC1 and PC2 variation, where negatively loaded bands have the strongest absorbance in positively scored samples and positively loaded bands will have the strongest absorbance in negatively scored samples.

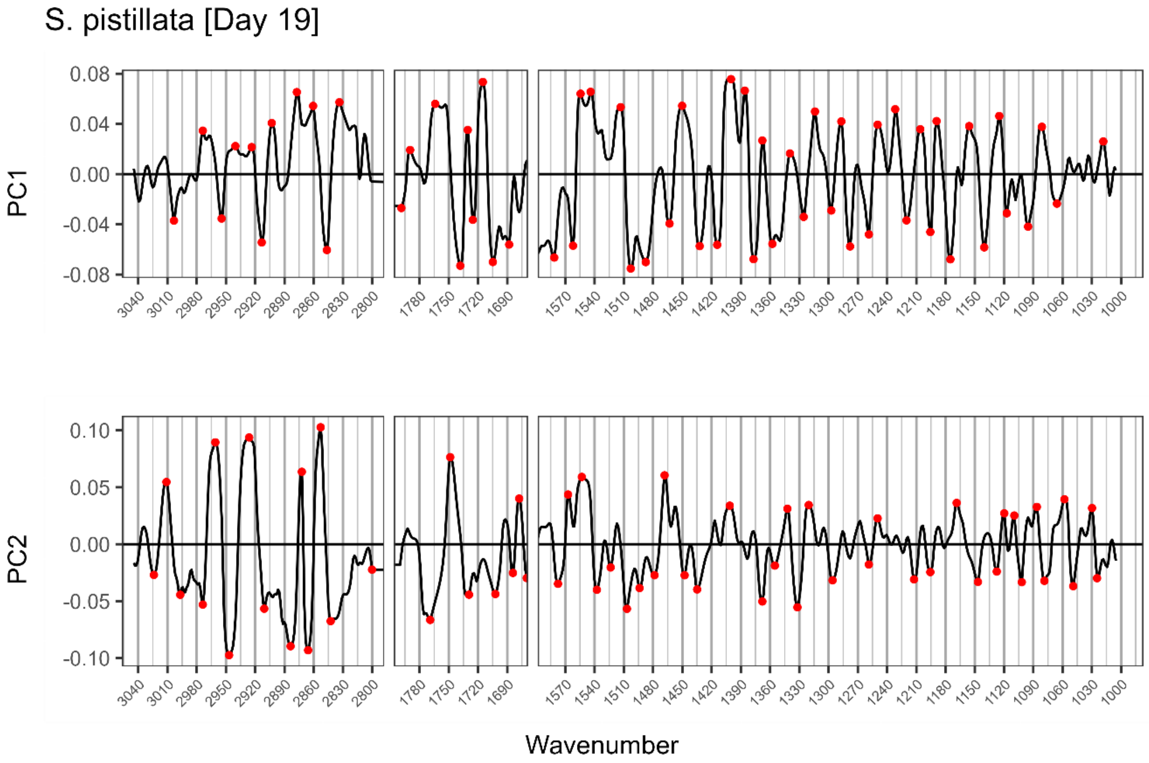

Figure S7. PCA loadings of *Stylophora pistillata* on day 19. Showing the contributions of each biomolecule to PC1 and PC2 variation, where negatively loaded bands have the strongest absorbance in positively scored samples and positively loaded bands will have the strongest absorbance in negatively scored samples.

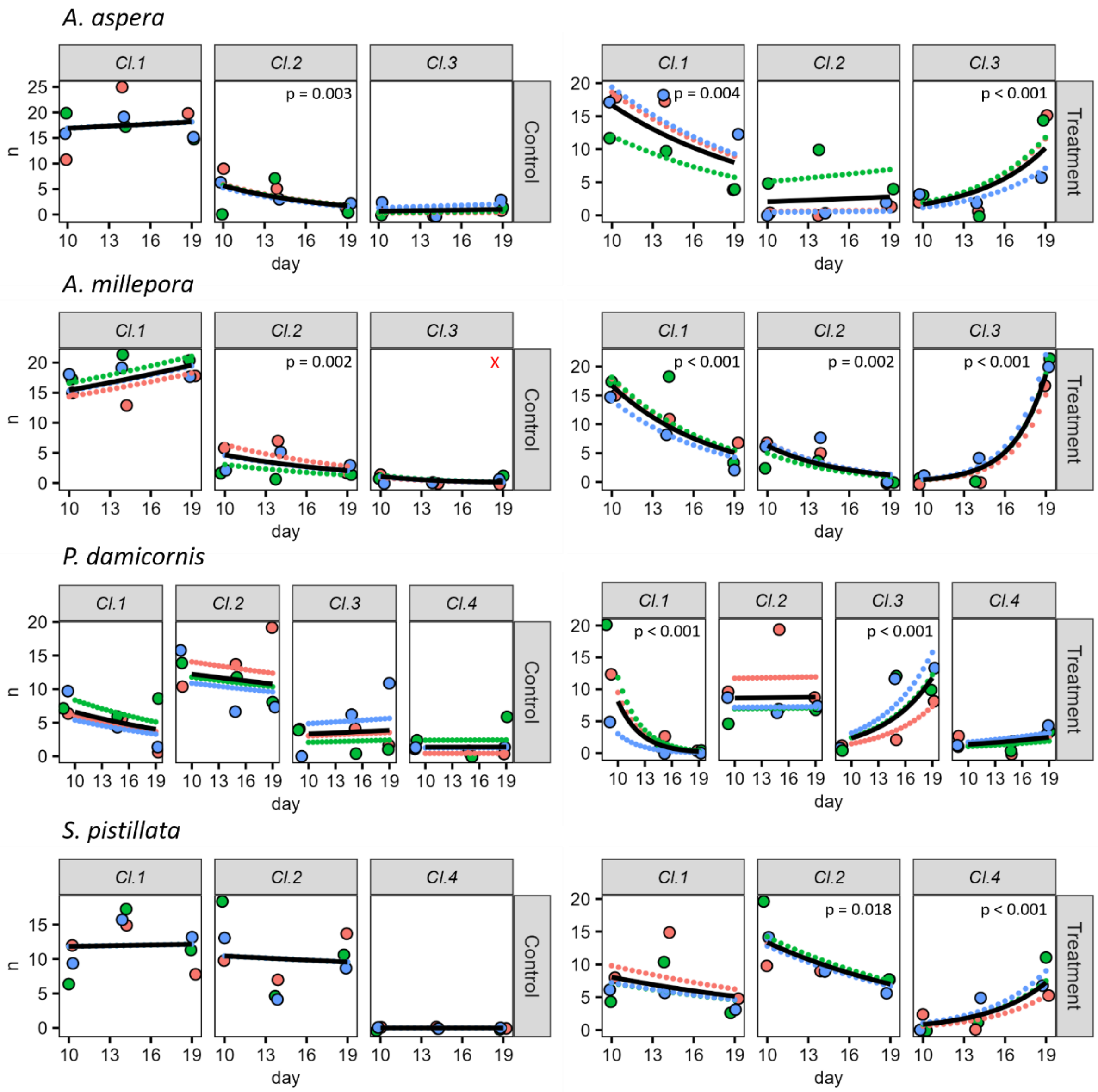

Figure S8. Generalized linear mixed-effects models (GLMM) for cluster prevalence in each coral colony for each species on each day and experimental condition. Dots indicate count of cells with specific cluster profile. Colours indicate colony. Stippled lines indicate model predictions for each colony. Black line indicate mean predicted value across colonies. Left: colonies kept under control conditions, right: colonies exposed to heat treatment. P values shown where model predicts significant change over time. Fitted model: n ~ day:cluster + (cluster|colony), family = "poisson". Red cross indicates data with zero-inflation which is not able to be suitably evaluated using this regression. See details of models and estimates in table S2.

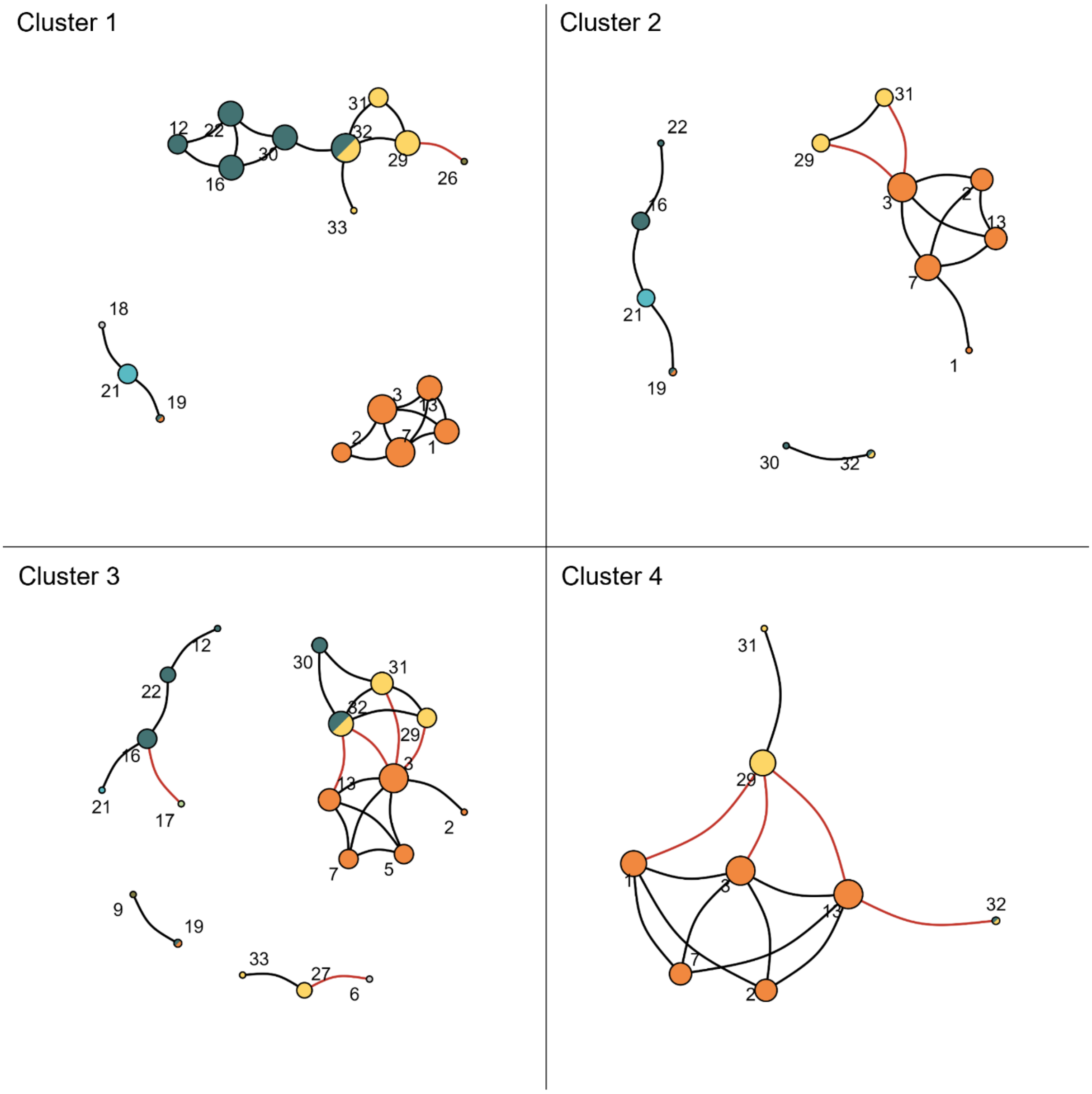

Figure S9. Network analysis of significant correlations across all species within a given cluster (p<0.001, rho >0.3). Note: the number of species represented differs by cluster: Clusters 1 & 2: n = 4; cluster 3: n = 3, cluster 4: n = 2. Edge colours: black indicates positive correlation, red indicates negative correlation. Size of nodes represents level of connectivity. Colour of nodes indicate compound group as per figure 4 in publication. Numbers indicate peak ID as per table 1 in publication.

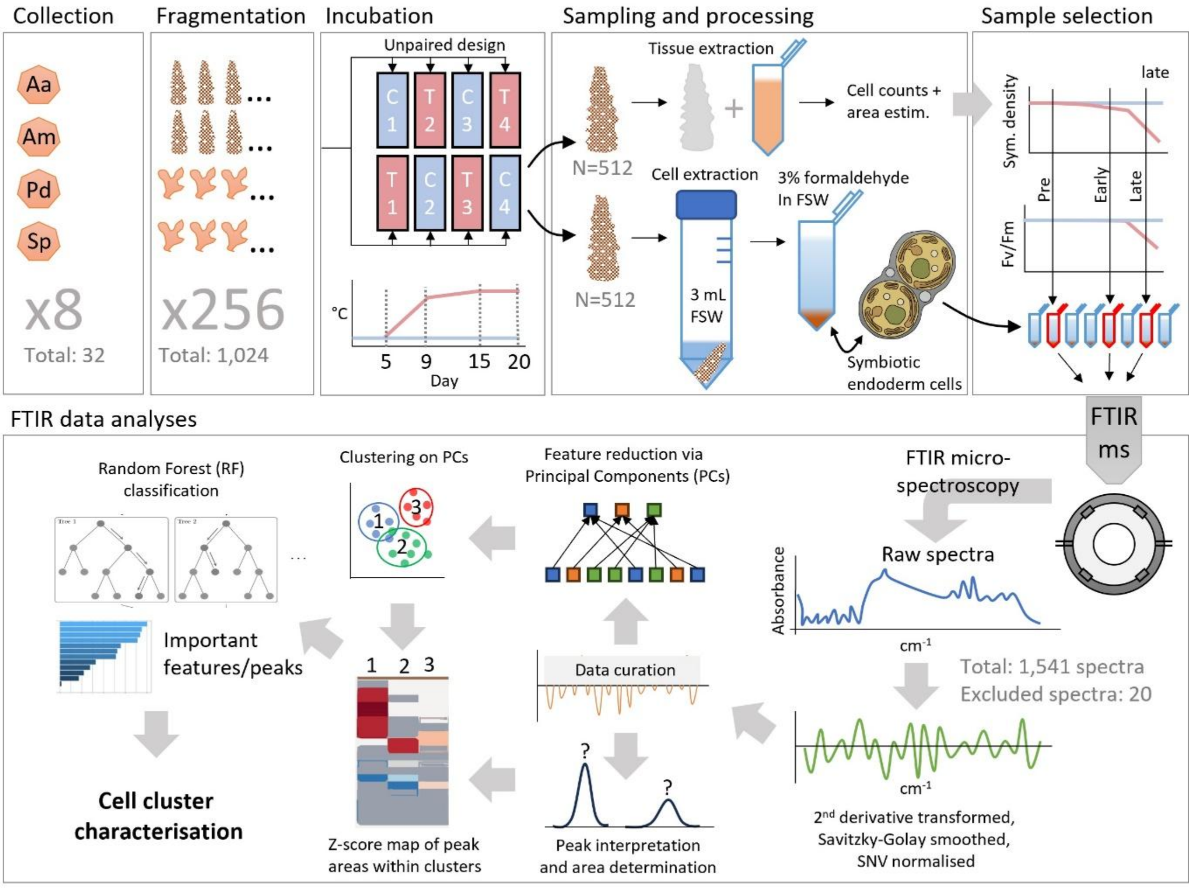

Figure S10. Overview of experimental design, sample selection and data analyses. Collection: Eight colonies of each species was collected. Fragmentation: Each colony was broken up into 32 fragments resulting in 256 fragments per species. Incubation: Fragments were assigned to one of eight tanks, four control tanks and four treatment tanks in which temperature was slowly increased to a mean of 31 degrees. Cells were sampled daily, and subsequent analyses were done on cells from selected days representing pre-, early and late stress. Selected samples were analysed using s-FTIR and spectra transformed before formal analyses.

Table S2: Output from Generalized Linear Mixed-Effects Models (negative binomial) for data presented in figure 4b and S8. Fitted model: n ~ day:cluster + (cluster|colony), family = "poisson". Where n is the count of cells in a given cluster on a given day. For simplicity, models were run on data pre-separated into treatment categories (control and treatment) and species.

***A. aspera* [Control]**

	**n**				
Predictors	*Log-Mean*	*std. Error*	*CI*	*Statistic*	*p*
(Intercept)	2.7491 ***	0.3134	2.1349 – 3.3633	8.7729	**<0.001**
day × clusterCl 1	0.0079	0.0207	-0.0327 – 0.0484	0.3798	0.704
day × clusterCl 2	-0.1322 **	0.0439	-0.2181 – -0.0462	-3.0133	**0.003**
day × clusterCl 3	0.0495	0.1137	-0.1734 – 0.2723	0.4351	0.664
N_{colony}	3				
Observations	27				

* *p<0.05* ** *p<0.01* *** *p<0.001*

***A, aspera* [Treatment]**

	n				
Predictors	*Log-Mean*	*std. Error*	*CI*	*Statistic*	*p*
(Intercept)	2.9581 ***	0.4586	2.0593 – 3.8570	6.4501	**<0.001**
day × clusterCl 1	-0.0815 **	0.0281	-0.1365 – -0.0265	-2.9018	**0.004**
day × clusterCl 2	0.0343	0.0589	-0.0812 – 0.1497	0.5817	0.561
day × clusterCl 3	0.2012 ***	0.0493	0.1046 – 0.2978	4.0815	**<0.001**
N_{colony}	3				
Observations	27				

* *p<0.05* ** *p<0.01* *** *p<0.001*

***M. millepora* [Control]**

	n				
Predictors	*Log-Mean*	*std. Error*	*CI*	*Statistic*	*p*
(Intercept)	2.4591 ***	0.2943	1.8824 – 3.0358	8.3571	**<0.001**

day × clusterCl 1	0.0266	0.0197	-0.0121 – 0.0653	1.3481	0.178
day × clusterCl 2	-0.0923 **	0.0294	-0.1500 – -0.0346	-3.1357	**0.002**
day × clusterCl 3	-0.2402 ***	0.0460	-0.3304 – -0.1499	-5.2162	**<0.001**
ICC	0.0723				
N colony	3				
Observations	27				
Marginal R^2 / Conditional R^2	0.973 / 0.975				

* p<0.05 ** p<0.01 *** p<0.001

***M. millepora* [Treatment]**

	n				
Predictors	*Log-Mean*	*std. Error*	*CI*	*Statistic*	*p*
(Intercept)	1.2280	2.0372	-2.7648 – 5.2208	0.6028	0.547
day × clusterCl 1	-0.1326 ***	0.0317	-0.1947 – -0.0704	-4.1815	**<0.001**
day × clusterCl 2	-0.1850 **	0.0591	-0.3009 – -0.0692	-3.1298	**0.002**
day × clusterCl 3	0.4270 ***	0.0675	0.2948 – 0.5592	6.3301	**<0.001**
ICC	0.9859				
N colony	3				
Observations	27				
Marginal R^2 / Conditional R^2	0.669 / 0.995				

* p<0.05 ** p<0.01 *** p<0.001

***P. damicornis* [Control]**

			**n**		
Predictors	*Log-Mean*	*std. Error*	*CI*	*Statistic*	*p*
(Intercept)	2.0980 ***	0.4753	1.1665 – 3.0296	4.4141	**<0.001**
day × clusterCl 1	-0.0547	0.0392	-0.1316 – 0.0222	-1.3945	0.163

day × clusterCl 2	-0.0142	0.0313	-0.0755 – 0.0471	-0.4539	0.650
day × clusterCl 3	0.0165	0.0514	-0.0843 – 0.1173	0.3205	0.749
day × clusterCl 4	0.0018	0.0774	-0.1498 – 0.1534	0.0233	0.981
ICC	0.4076				
N_{colony}	3				
Observations	36				
Marginal R^2 / Conditional R^2	0.303 / 0.587				

* *p<0.05* ** *p<0.01* *** *p<0.001*

***P. damicornis* [Treatment]**

	**n**				
Predictors	*Log-Mean*	*std. Error*	*CI*	*Statistic*	*p*
(Intercept)	1.0876	0.6804	-0.2460 – 2.4212	1.5984	0.110
day × clusterCl 1	-0.3949 ***	0.0763	-0.5445 – -0.2453	-5.1733	**<0.001**
day × clusterCl 2	0.0018	0.0275	-0.0521 – 0.0557	0.0658	0.948
day × clusterCl 3	0.1796 ***	0.0410	0.0994 – 0.2599	4.3855	**<0.001**
day × clusterCl 4	0.0680	0.0567	-0.0430 – 0.1791	1.2007	0.230
ICC	0.9939				
N_{colony}	3				
Observations	36				
Marginal R^2 / Conditional R^2	0.314 / 0.996				

* *p<0.05* ** *p<0.01* *** *p<0.001*

***S. pistillata* [Control]**

	n				
Predictors	*Log-Mean*	*std. Error*	*CI*	*Statistic*	*p*
(Intercept)	2.4425 ***	0.3378	1.7804 – 3.1047	7.2299	**<0.001**
day × clusterCl 1	0.0029	0.0234	-0.0430 – 0.0487	0.1221	0.903
day × clusterCl 2	-0.0097	0.0238	-0.0563 – 0.0369	-0.4078	0.683

day × clusterCl 4	-3.8603	-	-	-0.0000	1.000
ICC	0.0000				
N colony	3				
Observations	27				
Marginal R^2 / Conditional R^2	1.000 / 1.000				

* *p<0.05* ** *p<0.01* *** *p<0.001*

Note: Std Error and CI removed from S. pistillata control data cluster 4 due to nonsense model output.

***S. pistillata* [Treatment]**

	n				
Predictors	*Log-Mean*	*std. Error*	*CI*	*Statistic*	*p*
(Intercept)	1.7584	1.0708	-0.3403 – 3.8572	1.6421	0.101
day × clusterCl 1	-0.0499	0.0366	-0.1216 – 0.0218	-1.3648	0.172
day × clusterCl 2	-0.0719 *	0.0305	-0.1316 – -0.0121	-2.3572	**0.018**
day × clusterCl 4	0.2374 ***	0.0634	0.1131 – 0.3618	3.7426	**<0.001**
ICC	0.8355				
N colony	3				
Observations	27				
Marginal R^2 / Conditional R^2	0.842 / 0.974				

* *p<0.05* ** *p<0.01* *** *p<0.001*

Table S3: Output from linear mixed effect models on data presented in Figure 6b. Fitted model: value ~ cluster + (species|species/cluster) + (1|day) (REML = TRUE). All tests are against cluster 1. Exp_value represents the peak area (sqrt transformed or untransformed) of the respective peak.

Carbohydrate 5 (pyranose)

	exp_value				
Predictors	*Estimates*	*std. Error*	*CI*	*Statistic*	*p*
(Intercept)	0.9289 **	0.0444	0.6393 – 1.2185	20.9232	**0.010**
cluster [2]	-0.0403	0.0263	-0.0923 – 0.0116	-1.5329	0.127
cluster [3]	0.5013 ***	0.0305	0.4415 – 0.5611	16.4616	**<0.001**
cluster [4]	-0.1671 **	0.0542	-0.2736 – -0.0605	-3.0803	**0.002**
N_{colony}	3				
$N_{species}$	4				
Observations	1527				
Marginal R^2	0.209				

* *p<0.05* ** *p<0.01* *** *p<0.001*

Carboxylated molecules

	exp_value				
Predictors	*Estimates*	*std. Error*	*CI*	*Statistic*	*p*
(Intercept)	0.5315	0.1244	-0.0156 – 1.0786	4.2710	0.053
cluster [2]	0.3572 ***	0.0386	0.2813 – 0.4331	9.2502	**<0.001**
cluster [3]	0.7819 ***	0.0478	0.6882 – 0.8757	16.3616	**<0.001**
cluster [4]	0.9862 ***	0.0849	0.8196 – 1.1527	11.6222	**<0.001**
N_{colony}	3				
$N_{species}$	4				
N_{day}	5				
Observations	1529				
Marginal R^2	0.240				

* *p<0.05* ** *p<0.01* *** *p<0.001*

Protein (Amide II)

	exp_value				
Predictors	*Estimates*	*std. Error*	*CI*	*Statistic*	*p*
(Intercept)	1.1358 ***	0.0536	1.0027 – 1.2688	21.1905	**<0.001**
cluster [2]	-0.2100 ***	0.0125	-0.2346 – -0.1854	-16.7480	**<0.001**
cluster [3]	-0.3555 ***	0.0165	-0.3879 – -0.3231	-21.5169	**<0.001**
cluster [4]	-0.4925 ***	0.0278	-0.5470 – -0.4379	-17.7004	**<0.001**
N_{colony}	3				
$N_{species}$	4				
N_{day}	5				
Observations	1529				
Marginal R^2	0.387				

* *p<0.05* ** *p<0.01* *** *p<0.001*

Ester carbonyl

	exp_value				
Predictors	*Estimates*	*std. Error*	*CI*	*Statistic*	*p*
(Intercept)	0.9268 ***	0.0324	0.8459 – 1.0077	28.6367	**<0.001**
cluster [2]	0.0610 ***	0.0124	0.0367 – 0.0854	4.9150	**<0.001**
cluster [3]	0.1795 ***	0.0159	0.1483 – 0.2106	11.3087	**<0.001**
cluster [4]	0.1590 ***	0.0270	0.1061 – 0.2120	5.8926	**<0.001**
ICC	0.0343				
N_{colony}	3				
$N_{species}$	4				
N_{day}	5				
Observations	1514				
Marginal R^2	0.108				

* *p<0.05* ** *p<0.01* *** *p<0.001*

Saturated lipids CH-stretch IV

exp_value

Predictors	Estimates	std. Error	CI	Statistic	p
(Intercept)	0.9587 ***	0.0462	0.7971 – 1.1202	20.7507	**0.001**
cluster [2]	0.1559 ***	0.0093	0.1376 – 0.1742	16.7180	**<0.001**
cluster [3]	0.0600 ***	0.0123	0.0358 – 0.0842	4.8614	**<0.001**
cluster [4]	0.1860 ***	0.0208	0.1453 – 0.2267	8.9563	**<0.001**
N_{colony}	3				
$N_{species}$	4				
N_{day}	5				
Observations	1508				
Marginal R^2	0.209				

* $p<0.05$ ** $p<0.01$ *** $p<0.001$

Saturated fatty acids

			exp_value		
Predictors	*Estimates*	*std. Error*	*CI*	*Statistic*	*p*
(Intercept)	0.9647 ***	0.0265	0.8857 – 1.0437	36.3879	**<0.001**
cluster [2]	0.1253 ***	0.0111	0.1036 – 0.1470	11.3356	**<0.001**
cluster [3]	0.0260	0.0139	-0.0013 – 0.0532	1.8668	0.062
cluster [4]	0.0902 ***	0.0245	0.0421 – 0.1382	3.6824	**<0.001**
N_{colony}	3				
$N_{species}$	4				
N_{day}	5				
Observations	1500				
Marginal R^2	0.112				

* $p<0.05$ ** $p<0.01$ *** $p<0.001$

Unsaturated Fatty Acids

	exp_value				
Predictors	*Estimates*	*std. Error*	*CI*	*Statistic*	*p*
(Intercept)	1.0346 **	0.0347	0.8821 – 1.1871	29.8221	**0.001**
cluster [2]	-0.0600 ***	0.0133	-0.0860 – -0.0339	-4.5229	**<0.001**
cluster [3]	-0.1108 ***	0.0173	-0.1449 – -0.0767	-6.3884	**<0.001**
cluster [4]	-0.1622 ***	0.0289	-0.2189 – -0.1054	-5.6030	**<0.001**
N_{colony}	3				
$N_{species}$	4				
N_{day}	5				
Observations	1487				
Marginal R^2	0.057				

* *p<0.05* ** *p<0.01* *** *p<0.001*

References

1. Coleman AW. Biogeography and speciation in the pandorina/volvulina (chlorophyta) superclade. *Journal of Phycology*. 2001;**37**:836-51
2. LaJeunesse T. Diversity and community structure of symbiotic dinoflagellates from caribbean coral reefs. *Marine biology*. 2002;**141**:387-400
3. Schloss PD, Westcott SL, Ryabin T *et al.* Introducing mothur: Open-source, platform-independent, community-supported software for describing and comparing microbial communities. *Applied and Environmental Microbiology*. 2009;**75**:7537-41 https://doi.org/10.1128/aem.01541-09
4. Camacho C, Coulouris G, Avagyan V *et al.* Blast+: Architecture and applications. *BMC bioinformatics*. 2009;**10**:1-9
5. Eren AM, Morrison HG, Lescault PJ *et al.* Minimum entropy decomposition: Unsupervised oligotyping for sensitive partitioning of high-throughput marker gene sequences. *The ISME journal*. 2015;**9**:968-79
6. Tobin MJ, Puskar L, Barber RL *et al.* Ftir spectroscopy of single live cells in aqueous media by synchrotron ir microscopy using microfabricated sample holders. *Vibrational spectroscopy*. 2010;**53**:34-38
7. Bambery KR, Wood BR, McNaughton D. Resonant mie scattering (rmies) correction applied to ftir images of biological tissue samples. *Analyst*. 2012;**137**:126-32
8. Petrou K, Nielsen DA, Heraud P. Single-cell biomolecular analysis of coral algal symbionts reveals opposing metabolic responses to heat stress and expulsion. *Frontiers in Marine Science*. 2018;**5**:12 https://doi.org/10.3389/fmars.2018.00110

9. Author. Spectrum: Fast adaptive spectral clustering for single and multi-view data [Computer software]. Version R package version 1.1 https://CRAN.R-project.org/package=Spectrum. 2020.
10. Von Luxburg U. A tutorial on spectral clustering. *Statistics and computing*. 2007;**17**:395-416
11. Kuhn M. Building predictive models in r using the caret package. *Journal of Statistical Software*. 2008;**28**:1-26 https://doi.org/10.18637/jss.v028.i05
12. Author. Findpc: Find number of principal components in single-cell analysis [Computer software]. Version R package version 1.0. 2023.
13. Author. Umap: Uniform manifold approximation and projection [Computer software]. Version R package version 0.2.10.0 https://CRAN.R-project.org/package=umap. 2023.
14. Langfelder P, Horvath S. Wgcna: An r package for weighted correlation network analysis. *BMC Bioinformatics*. 2008;**9** https://doi.org/10.1186/1471-2105-9-559
15. Langfelder P, Horvath S. Fast r functions for robust correlations and hierarchical clustering. *Journal of Statistical Software*. 2012;**46**:1-17
16. Zhbankov R, Andrianov V, Marchewka M. Fourier transform ir and raman spectroscopy and structure of carbohydrates. *Journal of Molecular Structure*. 1997;**436**:637-54
17. Whelan DR, Bambery KR, Heraud P *et al.* Monitoring the reversible b to a-like transition of DNA in eukaryotic cells using fourier transform infrared spectroscopy. *Nucleic acids research*. 2011;**39**:5439-48
18. Cai S, Singh BR. Identification of β-turn and random coil amide iii infrared bands for secondary structure estimation of proteins. *Biophysical chemistry*. 1999;**80**:7-20
19. Kačuráková M, Wilson R. Developments in mid-infrared ft-ir spectroscopy of selected carbohydrates. *Carbohydrate polymers*. 2001;**44**:291-303
20. Omoike A, Chorover J. Spectroscopic study of extracellular polymeric substances from bacillus s ubtilis: Aqueous chemistry and adsorption effects. *Biomacromolecules*. 2004;**5**:1219-30
21. Zeroual W, Choisy C, Doglia SM *et al.* Monitoring of bacterial growth and structural analysis as probed by ft-ir spectroscopy. *Biochimica et Biophysica Acta (BBA)-Molecular Cell Research*. 1994;**1222**:171-78
22. Wong P, Wong RK, Caputo TA *et al.* Infrared spectroscopy of exfoliated human cervical cells: Evidence of extensive structural changes during carcinogenesis. *Proceedings of the National Academy of Sciences*. 1991;**88**:10988-92
23. Haaland DM, Jones HD, Thomas EV. Multivariate classification of the infrared spectra of cell and tissue samples. *Applied spectroscopy*. 1997;**51**:340-45
24. Colthup NB, Daly LH, Wiberley SE. *Introduction to infrared and raman spectroscopy*: Academic press New York, London, 1964.
25. Heraud P, Caine S, Sanson G *et al.* Focal plane array infrared imaging: A new way to analyse leaf tissue. *New Phytologist*. 2007;**173**:216-25
26. Barth A. Infrared spectroscopy of proteins. *Biochimica et Biophysica Acta (BBA)-Bioenergetics*. 2007;**1767**:1073-101

27. Bandekar J. Amide modes and protein conformation. *Biochimica et Biophysica Acta (BBA)-Protein Structure and Molecular Enzymology*. 1992;**1120**:123-43
28. Vongsvivut J, Heraud P, Gupta A *et al.* Ftir microspectroscopy for rapid screening and monitoring of polyunsaturated fatty acid production in commercially valuable marine yeasts and protists. *Analyst*. 2013;**138**:6016-31
29. Vongsvivut J, Heraud P, Gupta A *et al.* Synchrotron-ftir microspectroscopy enables the distinction of lipid accumulation in thraustochytrid strains through analysis of individual live cells. *Protist*. 2015;**166**:106-21
30. Vongsvivut J, Heraud P, Zhang W *et al.* Quantitative determination of fatty acid compositions in micro-encapsulated fish-oil supplements using fourier transform infrared (ftir) spectroscopy. *Food chemistry*. 2012;**135**:603-09
